# Gastric Metastasis of Primary Lung Cancer: Case Report and Systematic Review With Pooled Analysis

**DOI:** 10.3389/fonc.2022.922016

**Published:** 2022-07-08

**Authors:** Dong Tang, Jianjian Lv, Zhijing Liu, Shuhui Zhan, Yuqiang Gao

**Affiliations:** ^1^ Department of Gastroenterology, Qingdao Municipal Hospital, The Affiliated Municipal Hospital of Qingdao University, Qingdao, China; ^2^ Department of Oncology, Qingdao Municipal Hospital, The Affiliated Municipal Hospital of Qingdao University, Qingdao, China; ^3^ Department of Pathology, Qingdao Municipal Hospital, The Affiliated Municipal Hospital of Qingdao University, Qingdao, China

**Keywords:** gastric metastasis, primary lung cancer, EGFR mutation, clinicopathological features, prognosis

## Abstract

**Background:**

Gastric metastasis from lung cancer (GMLC) is a rare occurrence. The clinicopathological characteristics, outcomes, and prognostic factors remain largely elusive.

**Methods:**

We conducted a systematic review on case reports and case series of GMLC by scanning MEDLINE, Embase, and ISI Web of Knowledge. Data involving the clinicopathological features, treatment, and outcomes were extracted and analyzed. Survival analysis was performed using Kaplan–Meier method. The Cox proportional hazards regression model was used to identify potential prognostic factors associated with survival. Furthermore, a case of metastatic gastric adenocarcinoma of pulmonary origin with epidermal growth factor receptor (EGFR) L858R+T790M mutation was also described and included.

**Results:**

Seventy-eight records involving 114 cases (including ours) were finally included. The median age on admission was 65 years with a male predominance of 79.8%. Lung adenocarcinoma (42.1%), located in the right upper lobe (30.3%), was the most frequent primary tumor. Bleeding (36.7%) and abdominal pain (35.8%) were the two most common symptoms. Endoscopically, gastric lesions were typically presented as elevated lesions with or without volcano-like ulceration, or ulcerative lesions, mostly involving the gastric corpus. The median overall survival time and survival time after diagnosis of metastatic cancer were 11 months [95% confidence interval (CI): 7–14] and 4.5 months (95% CI: 3–9), respectively. The survival analyses revealed that surgical interventions (including lung surgery and/or abdominal surgery) and systemic therapy (including chemotherapy, radiotherapy, and/or targeted therapy) seemed to be positive prognostic factors for both overall survival and survival after diagnosis of metastatic cancer.

**Conclusions:**

Clinicians should be alerted to the occurrence of gastric metastasis in lung cancer patients. Comprehensive evaluation and appropriate treatment for specific patients may improve the survival rate of GMLC patients.

## Introduction

Lung cancer is a highly malignant tumor. About half of patients present metastasis at the time of diagnosis (1). The most common sites of extrapulmonary metastases are the liver, bone, brain, and adrenal glands (1). In very rare circumstances, lung cancer may metastasize to the stomach, the incidence of which has been reported to range from 0.19% to 5.1%, with a higher rate reaching 2%–14% in autopsy studies (2). Because of advances in the diagnosis and treatment of cancer, patients’ survival has gradually prolonged, making the encounter with gastric metastasis more frequent. However, only limited data have been published focusing on gastric metastasis from lung cancer (GMLC), and its clinical features and treatment strategy remained poorly understood. Especially when targeted therapies including epidermal growth factor receptor tyrosine kinase inhibitors (EGFR-TKIs) have been proven to induce a remarkable response in advanced non-small cell lung cancer (NSCLC) with EGFR-activating mutations (3), the effect of targeted therapies on GMLC patients has been barely reported. In the present study, we describe an unusual case of gastric metastasis from primary lung adenocarcinoma that was treated with the third-generation EGFR-TKI osimertinib and conduct a systematic review of previous case reports to study the clinical features, outcomes, and prognostic factors of this rare entity.

## Case Report

A 72-year-old man with a long-term smoking habit (one pack of cigarettes per day for 30 years) was referred to our hospital in April 2021 due to a 1-month history of recurrent fever and discovery of a right lung mass, which showed no change after antibiotic treatment.

His past medical history was significant for hypertension and diabetes mellitus for 5 years, and his medications were nifedipine gastrointestinal therapeutic system (GITS) 30 mg once daily, metformin 50 mg once daily, and acarbose 50 mg three times a day.

**Figure 1:**
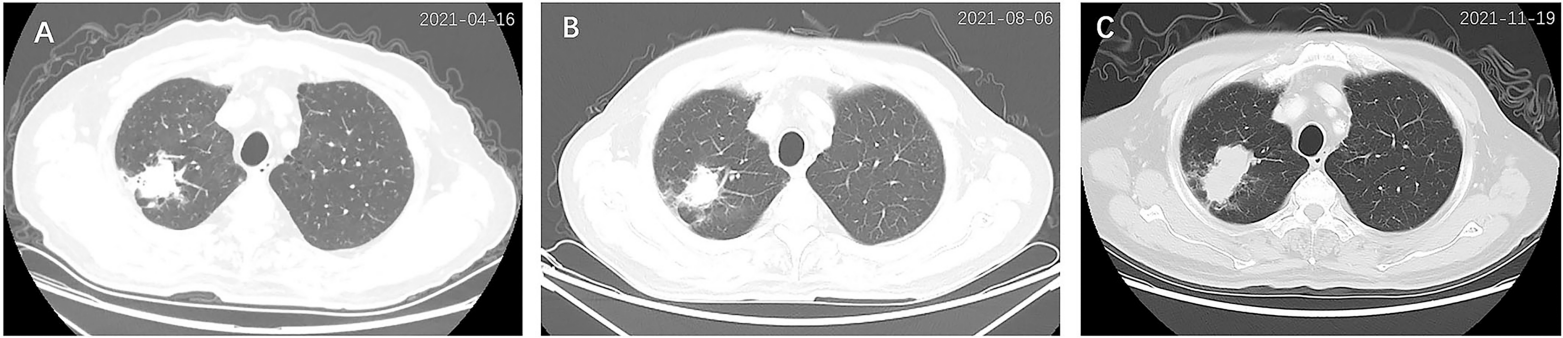
Chest computed tomography (CT) scan of the primary lung cancer at diagnosis **(A)**, 3 months after treatment **(B)**, and 6 months after treatment **(C)**. An irregular mass (3.5 cm × 2.7 cm) was detected in the right upper lobe **(A)**, which shrunk (2.6 cm × 2.2 cm) after 3 months of treatment **(B)** but enlarged (5.2 cm × 2.6 cm) after 6 months of treatment **(C)**.

**Figure 2:**
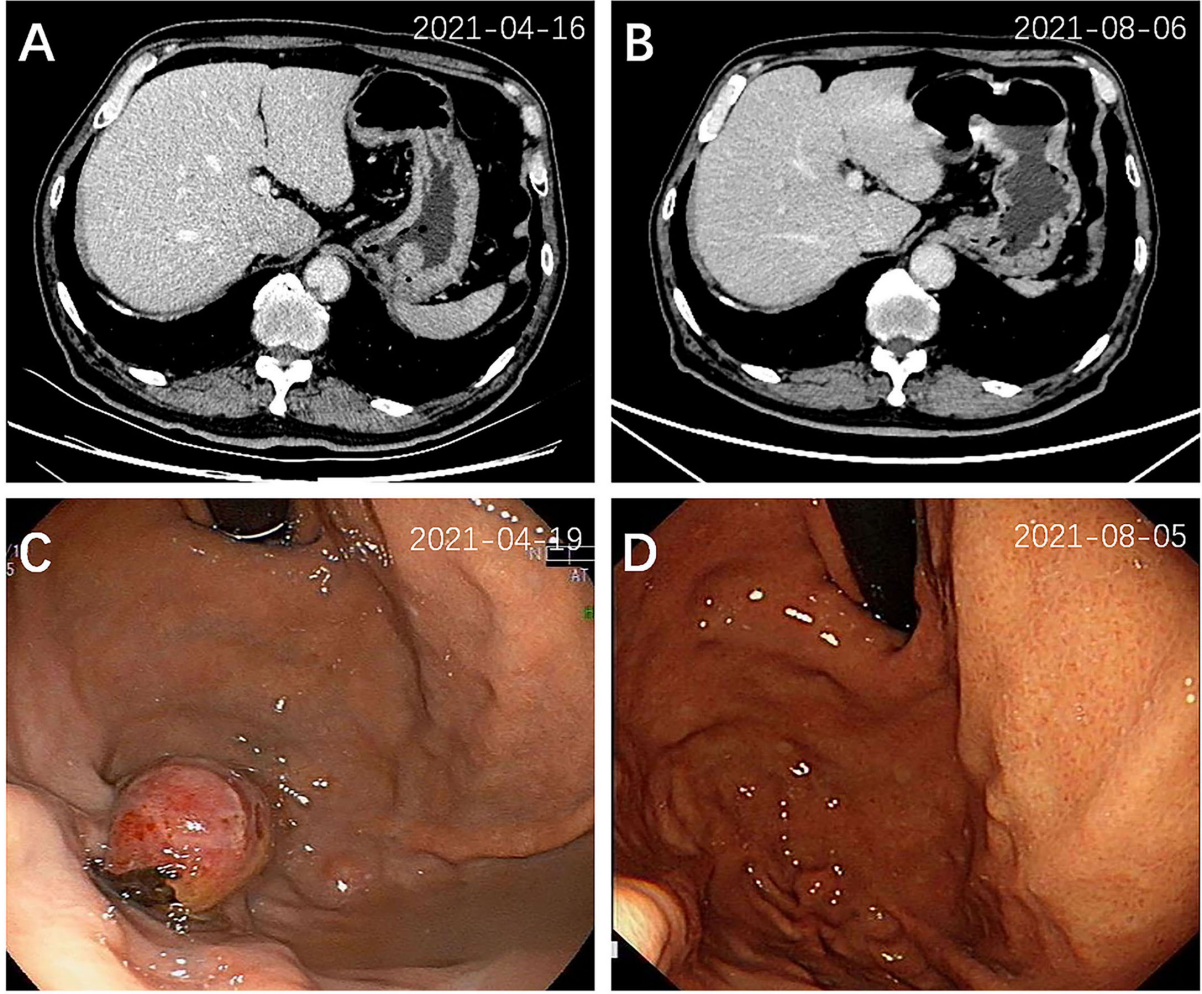
Abdominal CT scan and endoscopic view of the gastric tumor at diagnosis **(A, C)** and 3 months after treatment **(B, D)**. A mass (2.0 cm × 2.0 cm) located in the gastric fundus was detected by CT **(A)** and gastroscopy **(C)**, which disappeared 3 months after treatment **(B, D)**.

On admission, a computed tomography (CT) scan of the chest revealed an irregular mass measuring 3.5 cm × 2.7 cm in the right upper lobe (RUL), with mediastinal mildly enlarged lymph nodes (**Figure 1A**). Additional workup using abdominal CT detected a gastric fundal mass that measured 1.9 cm (**Figure 2A**). The patient denied any abdominal symptoms. Further gastroscopy demonstrated an ulcerated tumor 2.0 cm × 2.0 cm in size located in the gastric fundus (**Figure 2C**).

**Figure 3:**
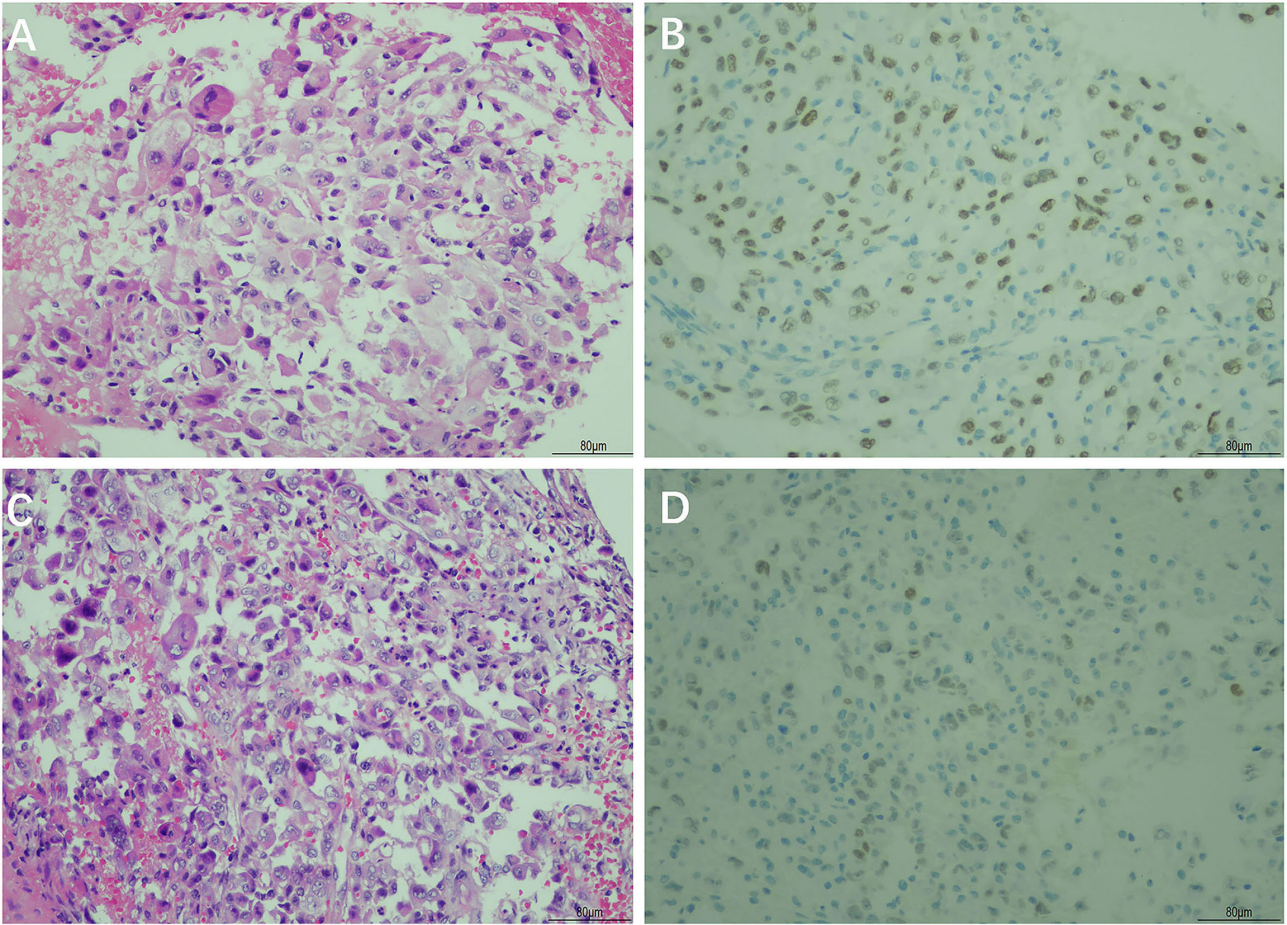
Hematoxylin and eosin (H&E) and immunohistochemical staining of the primary lung cancer and gastric tumor biopsy. H&E staining showed poorly differentiated adenocarcinoma in the primary lung cancer tissue **(A)** and gastric tumor tissue **(C)**. Immunohistochemical staining showed a positive reaction for thyroid transcription factor-1 (TTF-1) in the primary lung cancer tissue **(B)** and gastric tumor tissue **(D)** (magnification, ×200).

A CT-guided lung mass biopsy and pathological examination revealed poorly differentiated tumor cells (**Figure 3A**). The immunohistochemical stains showed that the tumor was thyroid transcription factor-1 (TTF-1) (+), CK7 (+), p63 (±), Napsin A (focally+), Ki67 (25%+), CK20 (-), CD56 (-), CK5/6 (-), and p40 (-), which is most consistent with lung adenocarcinoma (**Figure 3B**). Meantime, a gastric mass biopsy revealed poorly differentiated carcinoma with a similar morphological feature to the tumor from the pulmonary biopsy (**Figure 3C**). The immunohistochemical profile of the gastric sample showed TTF-1 (focally+), vimentin (+), Ki67 (40%+), CK7 (-), CK20 (-), Napsin A (-), p40 (-), CEA (-), villin (-), HER2 (-), and MOC31 (-) (**Figure 3D**). Furthermore, genetic studies demonstrated the same EGFR L858R+T790M mutation in both the gastric and pulmonary lesions, while the pulmonary sample also harbored a programmed cell death ligand 1 (PD-L1) Tumor Proportion Score (TPS) of 90%. All these findings supported the metastatic gastric adenocarcinoma of pulmonary origin. Additional brain CT and bone scan identified no abnormalities. The patient was diagnosed with poorly differentiated primary lung adenocarcinoma with gastric metastases (cT2N1M1 stage IV). Hence, oral treatment with osimertinib (80 mg, once a day) was started on May 13, 2021.

**Figure 4:**
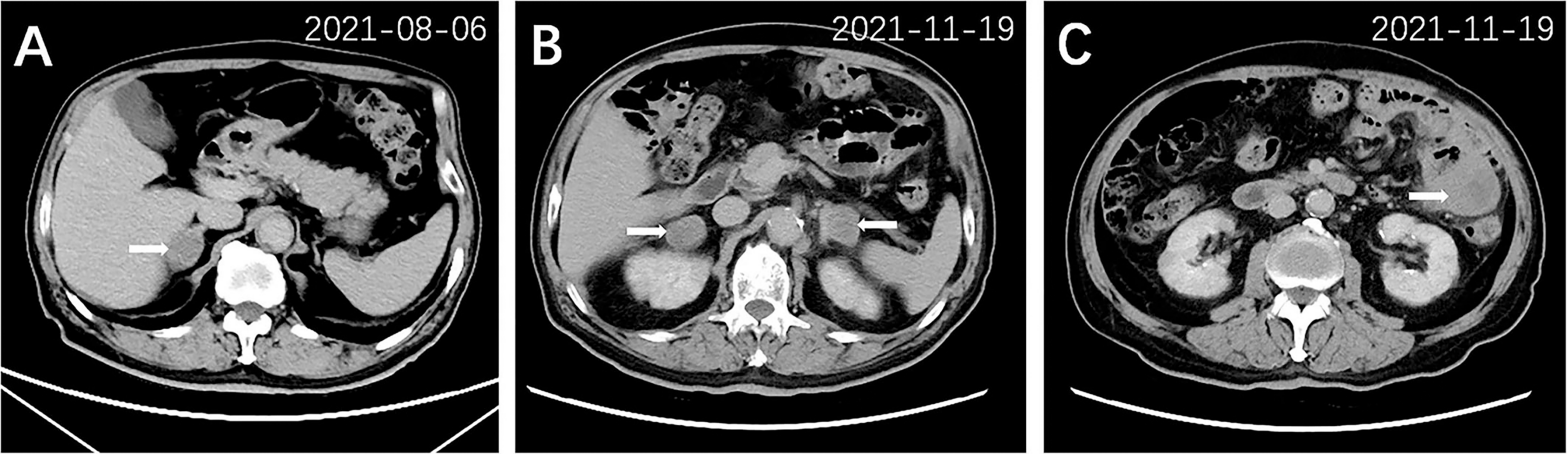
Abdominal CT scan of other metastatic lesions 3 months after treatment **(A)** and 6 months after treatment **(B, C)**. A nodule (2.9 cm × 2.0 cm) in the right adrenal gland (arrow) was detected after 3 months of treatment **(A)**. Multiple metastatic lesions were shown in bilateral adrenal glands (arrow, **B**) and left lower abdominal cavity (arrow, **C**) after 6 months of treatment.

After 3 months of treatment (August 2021), a follow-up chest CT scan revealed a reduction in the RUL mass (with the maximum cross section measuring 2.6 cm × 2.2 cm, **Figure 1B**). The gastric mass in the fundus exhibited complete regression in the CT scan (**Figure 2B**) and gastroscopy examination (**Figure 2D**). Meanwhile, an abdominal CT detected a nodule measuring 2.9 cm × 2.0 cm in the right adrenal gland, considered as a new metastatic lesion (**Figure 4A**). The patient’s primary lesion and gastric metastatic lesion were reduced, and a new adrenal gland metastasis was observed. According to RECIST 1.1 criteria (4), the efficacy was evaluated as progressive disease (PD). However, considering the effective treatment of primary lesions and gastric lesions, the patient chose to continue with osimertinib treatment. Unfortunately, 6 months after the initial diagnosis, the patient showed further disease progression with the enlargement of the primary lung mass (**Figure 1C**) and multiple metastatic lesions involving the bilateral adrenal glands and abdominal cavity (**Figures 4B, C**). The patient was recommended anti-PD-1 immunotherapy, multitargeting TKI (anlotinib), or chemotherapy. After communicating with the patient and his family, the patient opted for anlotinib treatment. At the time of writing, the patient is alive 8 months after the initial diagnosis of lung cancer.

## Systematic Review

### Methods

#### Search Strategy

A systematic review of the case reports was conducted to examine the clinical features and outcomes of GMLC. Literature search was performed by scanning MEDLINE (through PubMed), Embase, and ISI Web of Knowledge for relevant articles published until September 2021. The search terms included lung cancer-related and gastric metastasis-related index words. The specific search strategy is presented in the **Supplementary Material**. Reference lists of the relevant articles and reviews were carefully scanned to identify other eligible cases.

#### Study Selection

Two independent investigators (DT and JL) screened and included the relevant articles if they fulfilled all of the following criteria: 1) case reports or case series including the terms for gastric metastasis from primary lung cancer; 2) published in English or Chinese; and 3) provision of sufficient data on the demographic and/or clinicopathologic outcomes of GMLC cases. Articles were excluded if they were as follows: 1) reviews, meta-analysis, conference abstracts, or comment papers and 2) animal studies. Disparities were resolved with a third investigator (YG).

#### Data Extraction

Data such as title, author, publication year, age, gender, smoking habit, primary lung cancer site, pathological histology, interval time between the lung cancer diagnosis and gastric metastasis diagnosis, other metastasis site, clinical presentation, gastric tumor location, endoscopic appearance, treatment, and survival information were extracted by two investigators (JL and ZL) using a predefined form.

#### Statistical Analysis

Descriptive data were presented as median (interquartile range) and percentages. Overall survival (OS) was measured from the date of primary lung cancer diagnosis to the date of death. Survival after gastric metastasis was measured from the date of GMLC diagnosis to the date of death. Survival analysis was performed by Kaplan–Meier method. Univariate analysis was performed using Cox proportional hazards regression model, followed by a multivariate Cox regression analysis only including variables with a *P* value <0.10 during univariate analysis. Variables such as age, gender, number of metastases (solitary vs. multiple), interval (synchronous vs. metachronous), histology type, and treatment strategies were included in the univariate analysis. Synchronous metastasis is when the time interval of diagnosis between lung cancer and gastric metastasis was <1 month, while the time interval ≥1 month was considered as metachronous metastasis (5). Statistical analysis was performed using R software (version 4.0.3; The R Foundation for Statistical Computing, Vienna, Austria). A 2-sided *P* < 0.05 was considered statistically significant.

### Results

**Figure 5:**
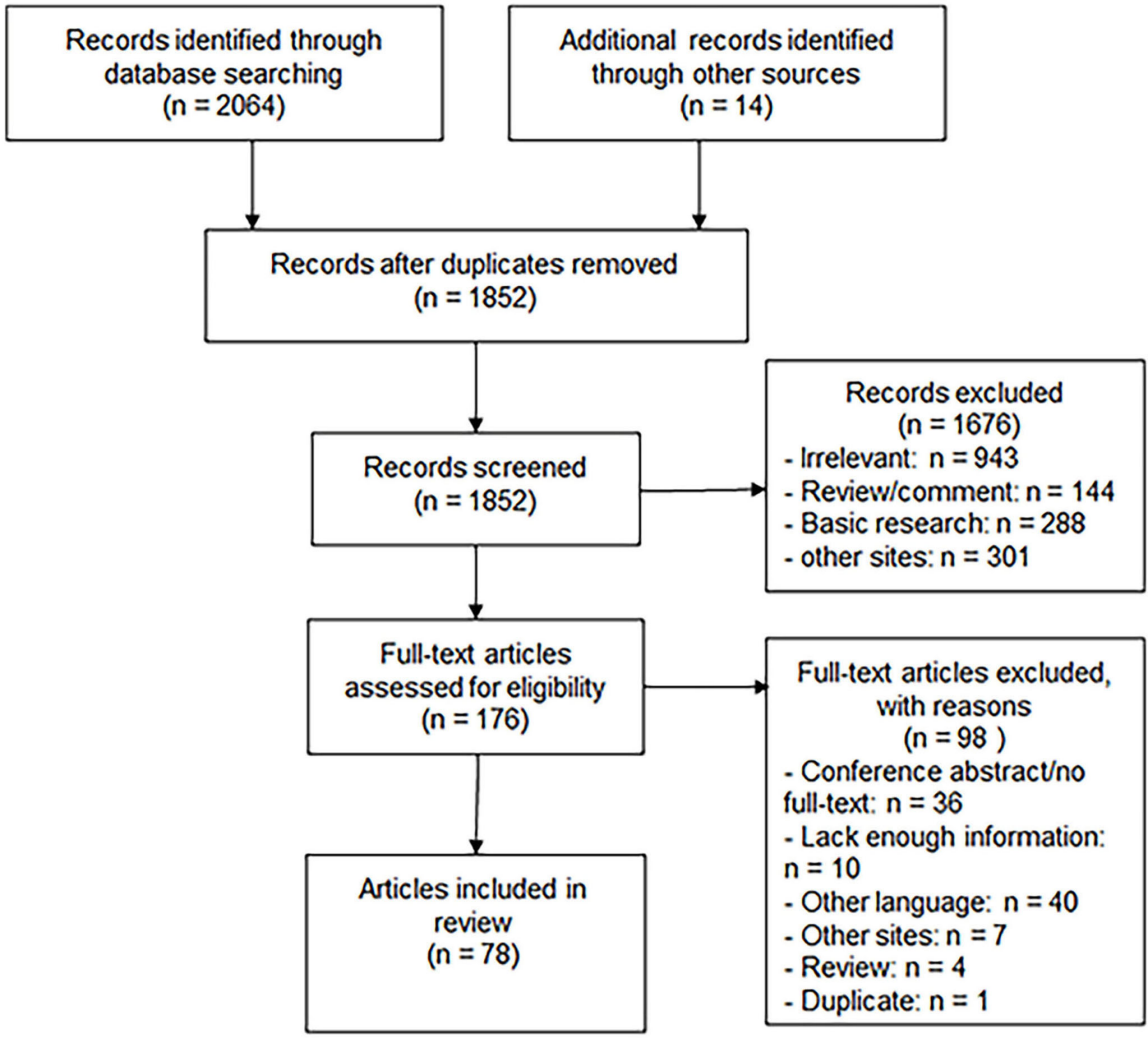
Flowchart of the selection process.

A total of 2,078 papers were retrieved, among which 2,064 were obtained through database search (PubMed: 260; Embase: 1,357; Web of Science: 447) and 14 through manual search. After 226 duplications and 1,676 papers were excluded by title and abstract screening, 176 were screened for full text and 78 papers were finally included in this systematic review, as shown in **Figure 5**.

A total of 114 cases were recruited in the present review (113 from the literature plus our case, **Table S1**) (2, 6–84). As shown in **Table 1**, the median age was 65 years (range, 59–71 years). There were 91 men (79.8%) and 23 women (20.2%). Among 54 cases that reported smoking habits, 42 patients (77.8%) were cigarette smokers, 12 (22.2%) had never smoked. NSCLC (99 cases, 86.8%) was the main histological type of GMLC. Adenocarcinoma (48 cases, 42.1%), squamous cell carcinoma (28 cases, 24.6%), and large cell lung cancer (12 cases, 10.5%) were the three most common histological types of NSCLC. Among 76 cases that reported the primary location of the lung cancer, gastric metastases were more commonly from the right lung (46 cases, 60.5%). Also, the most common site was the upper lobe (50%; right upper lobe: 30.3%; left upper lobe: 19.7%), followed by the lower lobe (23.7%; right lower lobe: 15.8%; left lower lobe: 7.9%) and hilum (18.4%; right hilum: 9.2%; left hilum: 9.2%).

**Table 1:** Demographic and clinicopathologic features of GMLC.

Characteristics	Value
**Age (years)**	65 (59–71)
**Gender**	
Men	91 (79.8)
Women	23 (20.2)
**Smoking** **(54 cases available)**	
Smoker	42 (77.8)
Non-smoker	12 (22.2)
**Histological type**	
Small cell lung cancer	15 (13.2)
Adenocarcinoma	48 (42.1)
Squamous cell carcinoma	28 (24.6)
Large cell carcinoma	12 (10.5)
Pleomorphic carcinoma	6 (5.3)
Primary lung sarcoma	2 (1.8)
Non-small cell lung cancer	3 (2.6)
**Primary lung location** **(76 cases available)**	
Right upper lobe	23 (30.3)
Right middle lobe	2 (2.6)
Right lower lobe	12 (15.8)
Right hilum	7 (9.2)
Right lung	2 (2.6)
Left upper lobe	15 (19.7)
Left lower lobe	6 (7.9)
Left hilum	7 (9.2)
Left lung	2 (2.6)
Both lung	1 (1.3)
**No. of metastasis sites** **(107 cases available)**	
Solitary	28 (26.2)
Multiple	79 (73.8)
**Interval** **(113 cases available)**	
Synchronous	54 (47.8)
Metachronous	59 (52.2)
**Interval time (m)** **(113 cases available)**	5 (1.6-13)

Data presented as the number of patients (%) or median (interquartile range).

GMLC, gastric metastasis from lung cancer.

In 107 cases that mentioned the number of metastatic sites, 28 cases (26.2%) presented as a single-site metastasis at the time of diagnosis, whereas 79 cases (73.8%) demonstrated other metastatic sites besides the stomach, with the liver, bone, brain, and adrenal gland being the four most prevalent metastatic sites. Moreover, 18 cases (16.8%) showed multiple metastases within the digestive tract, and the duodenum (11 cases, 10.3%) was the main concurrent site with the stomach, followed by the colon (4 cases, 3.7%, including 1 case that showed concurrent stomach, duodenum, and colon metastases), small intestine (3 cases, 2.8%), and esophagus (1 case, 0.9%). Synchronous (54 cases, 47.8%) and metachronous (59 cases, 52.2%) metastases demonstrated similar proportions. The median time between the primary lung cancer diagnosis and gastric metastasis diagnosis was 5 months (interquartile range, 1.6–13 months).

As presented in **Table 2**, bleeding was the most common symptom on admission, which was observed in 40 cases (36.7%; 21 melena; 4 hematemesis; 1 melena and hematemesis; 14 hemorrhage), followed by abdominal pain in 39 cases (35.8%) and anemia in 11 cases (10.1%). Eleven cases showed no symptoms (10.1%), and 3 cases (2.8%) presented with acute abdomen caused by perforation. Some cases also presented with abdominal discomfort, dysphagia, nausea, vomiting, or weight loss.

**Table 2:** Clinical and endoscopic features of gastric metastatic tumors.

Characteristics	Value
**Clinical presentation** **(109 cases available)**	
Bleeding	40 (36.7)
Abdominal pain	39 (35.8)
Anemia	11 (10.1)
Abdominal discomfort	5 (4.6)
Dysphagia	6 (5.5)
Nausea, vomiting	4 (3.7)
Weight loss	4 (3.7)
Perforation	3 (2.8)
Asymptomatic	11(10.1)
**Stomach location** **(89 cases available)**	
Corpus	50 (56.2)
Fundus	19 (21.3)
Antrum	13 (14.6)
Cardia	7 (7.9)
Whole	6 (6.7)
**Endoscopic appearance** **(85 cases available)**	
**Elevated lesions**	**50 (58.8)**
**Without ulcer**	**19 (22.4)**
SMT	8 (9.4)
Mass	2 (2.4)
Polypoidal mass	6 (7.1)
Nodules	3 (3.5)
**With ulcer**	**31 (36.5)**
SMT with ulcer (volcano-like)	13 (15.3)
Ulcerated mass (volcano-like)	15 (17.6)
Ulcerated nodules	3 (3.5)
**Ulcerated lesions**	**31 (36.5)**
Ulceration	12 (14.1)
Bulging ulcerated lesion (volcano-like)	10 (11.8)
Infiltrative ulcerated lesion	9 (10.6)
**Others**	**4 (4.7)**
*Linitis plastica*	3 (3.5)
Erosive and atrophic pangastritis	1 (1.2)

Data presented as the number of patients (%) or median (interquartile range).

SMT, submucosal tumor.

The bold value means the summarized patient numbers (bold numbers) and proportions (bold numbers in brackets) of relevant sub-items.

Metastatic lesions were mainly located in the corpus of the stomach (56.2% in 89 cases with whom the information regarding the metastatic site in the stomach was available), followed by fundus (19 cases, 21.3%), antrum (13 cases, 14.6%), and cardia (7 cases, 7.9%). Thirteen cases had lesions in two or more parts of the stomach.

According to the endoscopic appearance of gastric metastasis that was described in 85 cases, two main types of lesions were observed: the elevated lesions with or without ulceration (50 cases, 58.8%) and ulcerated lesions (31 cases, 36.5%). Moreover, elevated lesions with volcano-like ulceration were more common than that without ulceration (36.5% vs. 22.4%). Some cases also presented with pangastritis or *linitis plastica*-like features.

Immunohistochemical information was available in 58 cases, among which the typical immunophenotype of GMLC diagnosis was positive for TTF-1 (44, 75.9%), cytokeratin 7 (CK7, 31, 53.4%), and negative for CK20 (22, 37.9%), and caudal-related homeodomain transcription 2 (CDX2, 14, 24.1%). Other markers for diagnosis such as p63, CK5/6, CKAE1/AE3(+), and Napsin A were also reported.

As shown in **Table 3**, nearly one-third of cases underwent lung surgery (29.5%, mainly lobectomy) for primary lung cancer and abdominal surgery (31.5%, mainly partial or total gastrectomy) for gastric metastasis. Chemotherapy, radiotherapy, chemoradiotherapy, or targeted therapy were performed in 45.5% and 32.6% cases for primary lung cancer and gastric metastasis, respectively. Only supportive treatment was conducted in 25% and 35.9% of cases for primary lung cancer and gastric metastasis, respectively. The statistics were calculated based on cases with data available.

**Table 3:** Treatment and prognosis features of primary and metastatic tumors.

Characteristics	Value
**Primary lung treatment** **(88 cases available)**	
Lung cancer surgery	26 (29.5)
Non-surgery therapy	40 (45.5)
Supportive treatment	22 (25)
**Gastric metastasis treatment** **(92 cases available)**	
Abdominal surgery	29 (31.5)
Non-surgery therapy	30 (32.6)
Supportive treatment	33 (35.9)
**Survival information** **(93 cases available)**	
Dead	72 (77.4)
Alive	21 (22.6)
Survival after diagnosis of primary cancer, months	11 (7–14)
Survival after diagnosis of metastatic cancer, months	4.5 (3–9)

Data presented as the number of patients (%) or median (interquartile range).

Survival information was available for 93 cases. A total of 72 cases had succumbed to disease by the end of the study, and 21 cases were alive as reported, considered as censored data. The median OS was 11 months (95% CI: 7–14), with 1- and 3-year survival rates of 41.7% and 17.9%, respectively. The median survival time after diagnosis of metastatic cancer was 4.5 months (95% CI: 3–9), with 1- and 3-year survival rates of 24.9% and 10.5%, respectively.

**Figure 6:**
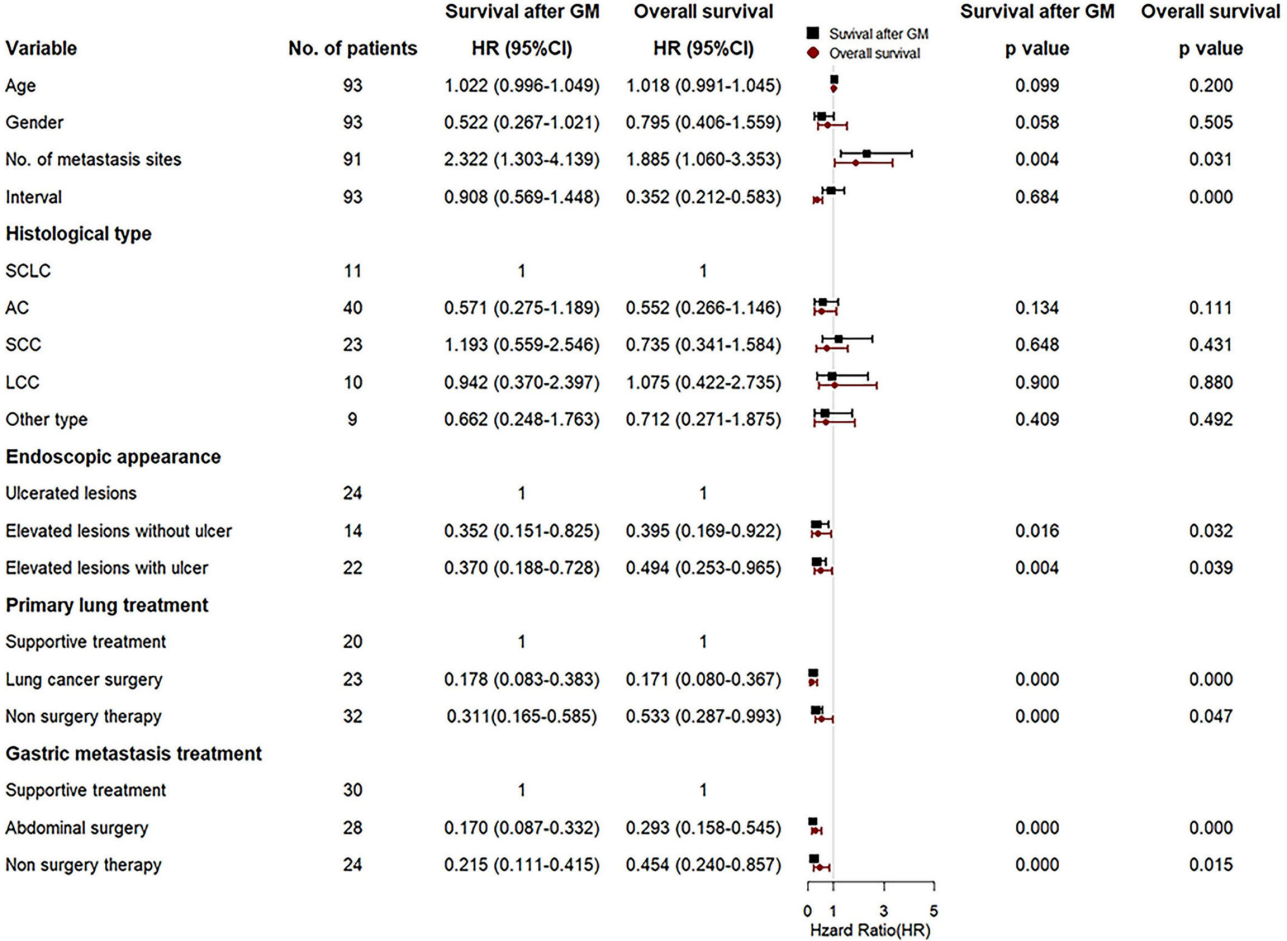
Forest plot for the univariate Cox regression analyses of variables that may affect survival after gastric metastasis and overall survival of the gastric metastasis from lung cancer (GMLC) patient.

As for survival after diagnosis of metastatic cancer, univariate Cox analysis revealed that cases with multiple metastatic sites exhibited poorer prognosis than that with solitary gastric metastasis [unadjusted hazard ratio (HR) 2.239, 95% CI: 1.255–3.992, *P* = 0.006], while cases manifested as elevated lesions with or without ulcer in the stomach (unadjusted HR 0.385, 95% CI: 0.195–0.760, *P* = 0.006; unadjusted HR 0.352, 95% CI: 0.150–0.825, *P* = 0.016, respectively) or that underwent surgery treatment for primary lung cancer or gastric metastasis lesions (unadjusted HR 0.178, 95% CI: 0.083–0.383, *P* = 0.000; unadjusted HR 0.171, 95% CI: 0.088–0.332, *P* = 0.000, respectively) or non-surgery therapy (unadjusted HR 0.321, 95% CI: 0.171–0.604, *P* = 0.000 for lung cancer; unadjusted HR 0.223, 95% CI: 0.116–0.432, *P* = 0.000 for gastric metastasis, respectively) demonstrated better outcomes compared with cases with ulcerated lesions in the stomach or underwent only supportive treatment (**Figure 6**). As for OS, similar prognostic factors were discovered, including synchronous, multiple metastasis, ulcerated lesions, supportive treatment that indicated poorer outcome, and metachronous, solitary metastasis, elevated lesions with or without ulcer, lung surgery, abdominal surgery and non-surgery therapy for gastric metastasis that indicated better survival prognosis (**Figure 6**).

**Figure 7:**
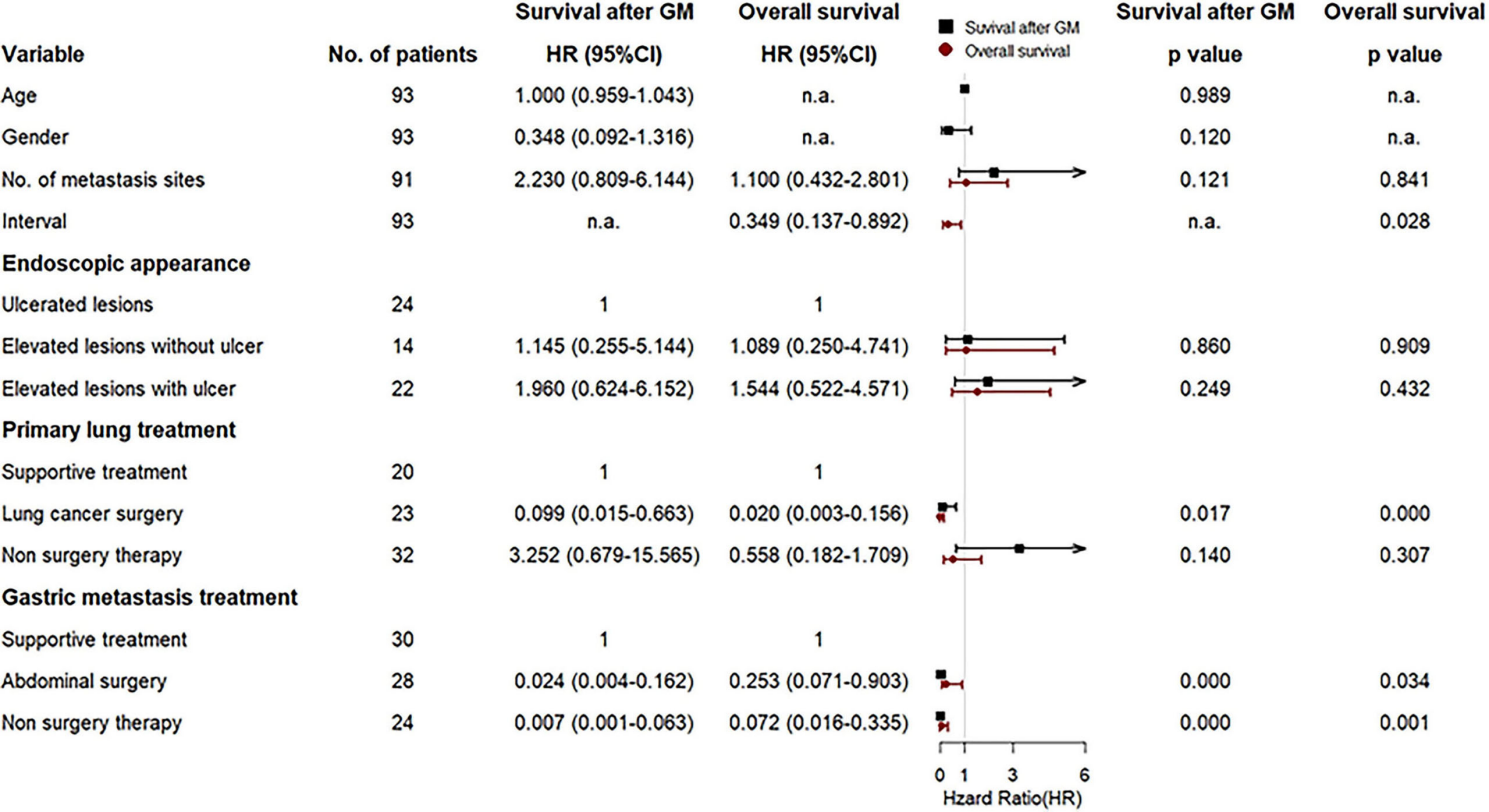
Forest plot for the multivariate Cox regression analyses of variables that may affect survival after gastric metastasis and overall survival of the gastric metastasis from lung cancer (GMLC) patient.

In multivariate Cox analysis, after adjustment for prognostic factors, lung surgery for primary lung cancer, abdominal surgery, and non-surgery therapy for gastric metastasis remained prognostic factors for both OS and survival after gastric metastasis, except for synchronous metastasis that indicated a prognostic factor only for OS (**Figure 7**). Other factors were not significant.

## Discussion

The occurrence of GMLC is rare. The diagnosis remains challenging especially when the primary lung cancer histology is adenocarcinoma. In this study, we described a case of gastric metastasis originating from lung adenocarcinoma, which was confirmed by tissue biopsy, immunohistochemistry, and mutational analysis. As the EGFR L858R+T790M mutations were detected, the patient was treated with the third-generation EGFR-TKI osimertinib but showing rapid disease progression. To our knowledge, our patient is probably the second reported case of lung cancer with gastric involvement treated with the new-generation EGFR-TKI (8). As there is difficulty in the diagnosis and treatment of gastric metastasis patients, we further systematically analyzed 114 GMLC cases to reveal the clinical features and prognostic factors of the patients.

In the present review, GMLC is more likely to occur in the old, and male is the more susceptible gender. Adenocarcinoma is the most frequent primary histological type resulting in gastric metastasis, which is consistent with previous reports (30, 49, 85–87). However, other certain studies have shown squamous cell carcinoma to be prominent (67, 88). Thus, the dominant primary histological type remains incompletely understood.

At present, the pathway underlying gastric metastasis is not clearly elucidated; however, hematogenous and lymphatic routes are supposed to be most likely involved in GMLC (15, 29, 85, 89). The metastatic tumor cells invade the submucosal layer through blood or lymph and develop into submucosal tumors (SMTs) (30, 34, 82), which remain clinically silent unless the gastric mucosa or serosa is involved or the tumor occupies the lumen (34, 53). Thus, most patients with GMLC are asymptomatic, and detection of gastric abnormality is usually by chance during follow-up or staging procedures of primary lung cancer, like that in our patient. When symptomatic, bleeding (mainly exhibited as melena) and abdominal pain were the two most common symptoms according to our review, all of which are nonspecific and usually misinterpreted as side effects of chemotherapy or indefinite complaints (30, 67, 75). Therefore, attention needs to be paid to gastrointestinal symptoms among lung cancer patients, and endoscopic examination is recommended for further evaluation.

Endoscopically, metastatic lesions most commonly present as a solitary ulcerated lesion located in the gastric corpus (86). The typical morphological appearance has been reported as SMT-like masses with elevation and ulceration at the apex, so-called “volcano-like’’ lesions (90, 91). Some lesions also appear as ulcers, polypoid nodules, or thickened walls (29). However, these endoscopic features are nonspecific, and differential diagnosis with primary lesions such as primary gastric cancer (GC) and lymphoma should be considered (14). Furthermore, about 9.4% of GMLC lesions manifested as SMTs with intact overlying mucosa, making conventional endoscopic biopsies frequently inconclusive. Endoscopic ultrasonography (EUS) is thus recommended for further evaluation. In EUS images, the metastatic tumors generally appeared as slightly hypoechoic lesions (more hyperechoic than the muscular tissue) involving the muscularis propria (fourth layer), mimicking primary subepithelial lesions such as gastrointestinal stromal tumors (GISTs), leiomyomas, and schwannomas (92, 93). EUS-guided fine-needle aspiration and biopsy (EUS-FNA/B) is currently the gold standard tissue sampling method for SMTs (92, 93). Hence, biopsies or EUS-FNA/B in conjunction with immunohistochemistry provides a reliable method to identify metastatic gastric tumors.

Several immunohistochemical markers have been reported to be useful for subclassifying tumors of different types and sites, such as TTF-1, Napsin A for lung adenocarcinoma, CDX2 for intestinal-type adenocarcinoma, and p63, CK5/6, CK34βE12/CK903 for squamous cell carcinoma (SCC) (14, 94, 95). Currently, TTF-1 is the most widely used stain for adenocarcinomas of pulmonary origin, with 61.5% sensitivity and 100% specificity in a series of 34 primary and metastatic adenocarcinomas in the lung (96). Also, different expression patterns of CK7 and CK20 are helpful for distinguishing tumor origin, with CK7+/CK20- for primary lung cancer and CK7-/CK20+ for gastrointestinal cancer (41, 62). Thus, a marker panel composed of TTF-1, CK7, CK20, and CDX-2 may be recommended to determine whether a gastric tumor was a primary or a pulmonary metastasis.

At present, there is no standard treatment protocol for GMLC patients, and treatment should be personalized according to pathology and patients’ condition. The therapeutic strategy includes surgery, chemotherapy with or without radiotherapy, targeted therapies, and supportive treatment (14).

Generally, the presence of a distant metastasis is a contraindication for surgery. High perioperative mortality and poor outcomes had been observed in surgical gastric and/or duodenal metastatic patients (49). However, our study and some other reports showed that surgery seemed to be a positive prognostic factor for GMLC patients (5, 30, 50). Accordingly, patients with solitary gastric metastasis may exhibit a survival benefit with surgical intervention (29, 50). Also, surgery may be necessary to prevent and/or control life-threatening complications such as massive hemorrhage or perforation (14, 29). Therefore, we considered surgery an option to treat gastric metastasis in properly selected patients, such as patients with unique metastatic lesions in the stomach and generally good condition, or with uncontrolled severe complications. With respect to radical surgery for isolated gastric metastasis, the optimal operating method remains to be clarified. According to our review, among 29 cases that underwent gastric surgical intervention, 5 cases received total gastrectomy, while 14 received partial or subtotal gastrectomy. The extent of gastric resection may depend on the site and size of the tumor. In selected GC patients such as early-stage and distal-third GC, subtotal gastrectomy may provide similar survival rates and better functional outcome compared to total gastrectomy (97). More recently, function-preserving gastrectomies such as proximal gastrectomy and pylorus-preserving gastrectomy have shown the advantages of preserving partial gastric physiologic functions and improving postoperative quality of life while maintaining radicality in early GC patients (98, 99). However, the impact of different surgical strategies including total gastrectomy, subtotal gastrectomy, or function-preserving gastrectomy on isolated metastatic gastric lesions still remains unclear and needs further investigation. In the present case, given the old age and generally poor condition, oral targeted therapy was prescribed other than surgery.

Currently, EGFR-TKIs represent the standard of care for advanced NSCLC patients with activating EGFR mutations, with median progression-free survival (PFS) ranging from 10 to 14.7 months (100). However, the efficacy of EGFR-TKIs on NSCLC with gastric metastasis has been barely reported. According to the present review, three cases were detected with the EGFR exon 19 deletions in gastric metastasis (24, 25, 30), which is the most common EGFR-TKI-sensitive activating mutation (101), and were treated with first-generation EGFR-TKI erlotinib. All of them tolerated the treatment well and were alive at the time of writing the reports (24, 25, 30). Our case harbored both L858R and T790M mutation at diagnosis, the latter of which is perceived as the most common resistance mutation associated with first- and second-generation EGFR-TKIs (101). At present, for NSCLC patients with T790M mutation, the third-generation EGFR-TKI osimertinib is recommended (100, 101). Also, in a randomized phase III FLAURA trial, osimertinib as first-line treatment exhibited improved PFS (18.9 months) and OS (38.6 months) compared with first-generation EGFR TKIs (median PFS of 10.2 months; median OS of 31.8 months) (102, 103). Therefore, our case was started on first-line therapy with oral osimertinib. Nevertheless, the patient experienced disease progression after 3 months of treatment, although the lesions of the lung and stomach exhibited partial response. The reason for the poor response to osimertinib in our case remains unclear. The reported potential mechanisms of resistance to osimertinib include the emergence of on-target resistance mutation such as EGFR C797S, bypass pathway activation such as MET amplification, or histologic small cell transformation (8, 100, 101, 104). Timely rebiopsies with comprehensive genomic profiling following disease progression on osimertinib therapy may be helpful for unraveling the resistance mechanisms (8). The effective therapies after osimertinib resistance still remain elusive. Chemotherapy, immunotherapy, and antiangiogenic therapy, either alone or in combination, may be considered for further treatment (100). Also, the combination of EGFR-TKIs with other therapeutic agents such as chemotherapy or vascular endothelial growth factor (VEGF) inhibitors has emerged as a potential therapeutic approach in the first-line setting to overcome EGFR-TKI resistance (101, 104). Several clinical trials are currently exploring the role of combination approaches with osimertinib (105), which may provide critical information to inform future treatment practice.

In summary, GMLC is a rare entity with poor prognosis. Diagnosis can be challenging as for the nonspecific symptoms and heterogeneous endoscopic appearances. Histological examination with immunohistochemical staining may help to confirm the diagnosis, and genomic profiling may provide valuable information for the diagnosis and therapeutic options. Treatment should be personalized, with surgery and systemic therapy (chemotherapy, radiotherapy, and/or targeted therapy) demonstrating better survival prognosis than only supportive care. The new-generation EGFR TKI osimertinib, either alone or combined with other therapeutic agents, emerges as a promising therapeutic strategy for metastatic NSCLC patients with EGFR-activating mutations. However, more clinical evidence is needed for exploring the efficacy of osimertinib on GMLC patients.

## Data Availability Statement

The original contributions presented in the study are included in the article/**Supplementary Material**. Further inquiries can be directed to the corresponding author.

## Author Contributions

YG conceived the idea and designed the study. DT collected clinical data, conducted the literature search, and analyzed the literature data. JL collected clinical data, performed the follow up, conducted the literature search, and extracted the literature data. ZL collected pathological data, and extracted the literature data. DT wrote the first version of manuscript. SZ revised the article. All authors have read and approved the final manuscript.

## Conflict of Interest

The authors declare that the research was conducted in the absence of any commercial or financial relationships that could be construed as a potential conflict of interest.

## Publisher’s Note

All claims expressed in this article are solely those of the authors and do not necessarily represent those of their affiliated organizations, or those of the publisher, the editors and the reviewers. Any product that may be evaluated in this article, or claim that may be made by its manufacturer, is not guaranteed or endorsed by the publisher.

## Supplementary Material

Supplementary Figure 1 Kaplan–Meier plot of the survival after gastric metastasis and overall survival curve.

## References

[1] Arbour KC Riely GJ . Systemic Therapy for Locally Advanced and Metastatic Non-Small Cell Lung Cancer: A Review. Jama (2019) 322(8):764–74. doi: 10.1001/jama.2019.11058 31454018

[2] Duan X Zhao X Wang S . An Alk-Positive Lung Adenocarcinoma With Gastric and Skin Metastasis: A Case Report and Literature Review. Ann Palliative Med (2021) 10(5):5797–807. doi: 10.21037/apm-20-1025 32819126

[3] Greenhalgh J Boland A Bates V Vecchio F Dundar Y Chaplin M . First-Line Treatment of Advanced Epidermal Growth Factor Receptor (Egfr) Mutation Positive Non-Squamous Non-Small Cell Lung Cancer. Cochrane Database System Rev (2021) 3(3):Cd010383. doi: 10.1002/14651858.CD010383.pub3 33734432 PMC8092455

[4] Eisenhauer EA Therasse P Bogaerts J Schwartz LH Sargent D Ford R . New Response Evaluation Criteria in Solid Tumours: Revised Recist Guideline (Version 1.1). Eur J Cancer (Oxford Engl 1990) (2009) 45(2):228–47. doi: 10.1016/j.ejca.2008.10.026 19097774

[5] Hu Y Feit N Huang Y Xu W Zheng S Li X . Gastrointestinal Metastasis of Primary Lung Cancer: An Analysis of 366 Cases. Oncol Lett (2018) 15(6):9766–76. doi: 10.3892/ol.2018.8575 PMC6004691 29928351

[6] Shih-Chun C Shih-Chiang H Chun-Yi T Shan-Yu W Keng-Hao L Jun-Te H . Non-Small Cell Lung Cancer With Gastric Metastasis and Repeated Gastrointestinal Bleeding: A Rare Case Report and Literature Review. Thorac Cancer (2021) 12(4):560–3. doi: 10.1111/1759-7714.13815 PMC7882379 33403816

[7] Sgaramella LI Gurrado A Fischetti E De Luca GM Pasculli A Brascia D . Rare Gastrointestinal Metastases From Primary Lung Cancer. Two Case Reports of Patients Managed With Emergency Surgery. Annali italiani di chirurgia (2021) 92:155–61. 34031285

[8] Liu J Xia L Peng Y Huang YS Yang ZZ . Gastric Metastasis and Transformation of Primary Lung Adenocarcinoma to Small Cell Cancer After Acquired Resistance to Epidermal Growth Factor Receptor Tyrosine Kinase Inhibitors: A Case Report. Medicine (2021) 100(39):e27289. doi: 10.1097/md.0000000000027289 34596125 PMC8483845

[9] Eiswerth MJ Pinter A Reynolds SB Guardiola J . Primary Lung Sarcoma With Gastric Metastasis and Morphological Divergence Presenting as Melena. BMJ Case Rep (2021) 14(8) :e242364. doi: 10.1136/bcr-2021-242364 PMC8362720 34385220

[10] Nemoto M Prasoon P Ichikawa H Hanyu T Kano Y Muneoka Y . Primary Lung Squamous Cell Carcinoma and Its Association With Gastric Metastasis: A Case Report and Literature Review. Thorac Cancer (2020) 11(6):1708–11. doi: 10.1111/1759-7714.13410 PMC7262906 32212371

[11] Peng Y Liu Q Wang Y Song A Duan H Qiu Y . Pathological Diagnosis and Treatment Outcome of Gastric Metastases From Small Cell Lung Cancer: A Case Report. Oncol Lett (2019) 18(2):1999–2006. doi: 10.3892/ol.2019.10484 31423270 PMC6607122

[12] He Y Cui Y Duan X Liu C Cai X . Primary Lung Squamous Cell Carcinoma With Gastric Metastasis: A Case Report. Thorac Cancer (2019) 10(2):373–7. doi: 10.1111/1759-7714.12940 PMC6360227 30561123

[13] Yang X Chen R Wu C Zhao W Ji M . Mutational Analysis on Gastric, Duodenal, Bone, and Mediastinal Lymph Node Metastases and Blood From a Case of Primary Lung Adenocarcinoma. OncoTargets Ther (2018) 11:4029–34. doi: 10.2147/OTT.S167602 PMC6049053 30034242

[14] Nitipir C Ginghina O Popa L Andrei F Tudor N Radu I . A Rare Case of Advanced Lung Cancer Presenting as a Symptomatic Gastric Tumor. Mol Clin Oncol (2018) 8(4):595–9. doi: 10.3892/mco.2018.1565 PMC5838308 29541469

[15] Li X Li S Ma Z Zhao S Wang X Wen D . Multiple Gastrointestinal Metastases of Squamous-Cell Lung Cancer: A Case Report. Medicine (2018) 97(24):e11027. doi: 10.1097/md.0000000000011027 29901596 PMC6023823

[16] El Hajj II Lawrence KA Tirkes T Shahda S Sherman S . Metachronous Gastric Metastasis From Lung Primary, With Synchronous Pancreatic Neuroendocrine Carcinoma. Clin Case Rep (2018) 6(7):1368–70. doi: 10.1002/ccr3.1571 PMC6028405 29988660

[17] Zhang B Xie L Yao Y Jing J . Gastric Metastasis of Primary Lung Adenocarcinoma: Report of Two Cases and Review of Literature. Cancer Res Clinic (2017) 29(8):563–4. doi: 10.3760/cma.j.issn.1006-9801.2017.08.015

[18] Taira N Kawabata T Gabe A Furugen T Ichi T Kushi K . Analysis of Gastrointestinal Metastasis of Primary Lung Cancer: Clinical Characteristics and Prognosis. Oncol Lett (2017) 14(2):2399–404. doi: 10.3892/ol.2017.6382 PMC5530210 28781676

[19] Sharma P Dwary AD Khan EM . Serendipitous Discovery of Isolated Gastric Metastases From Adenocarcinoma of the Lung on Staging 18f-Fdg Pet-Ct. Clin Nucl Med (2017) 42(10):807–8. doi: 10.1097/rlu.0000000000001784 28806259

[20] Qasrawi A Ghanimeh MA Albadarin S Yousef O . Gastric Metastases From Lung Adenocarcinoma Causing Gastrointestinal Bleeding. ACG Case Rep J (2017) 4(4):e25. doi: 10.14309/crj.2017.25 28286791 PMC5340659

[21] Bhardwaj R Bhardwaj G Gautam A Karagozian R . Upper Gastrointestinal Bleed as a Manifestation of Poorly Differentiated Metastatic Squamous Cell Carcinoma of the Lung. J Clin Diagn Res JCDR (2017) 11(6):Od13–od4. doi: 10.7860/jcdr/2017/27040.10090 PMC5535421 28764229

[22] Azar I Koutroumpakis E Patel R Mehdi S . Squamous Cell Lung Carcinoma Presenting as Melena: A Case Report and Review of the Literature. Rare Tumors (2017) 9(3):85–8. doi: 10.4081/rt.2017.7164 PMC5658634 29085618

[23] Pan X Chen LX Xu XY Zhou GR Yu SR Xie C . Response to Egfr-Tki in Patients With Gastrointestinal Metastasis From Primary Lung Adenocarcinoma: Report of Two Cases. Int J Clin Exp Pathol (2016) 9(5):5780–6.

[24] Ding LY Liu KJ Jiang ZL Wu HY Wu SX . Targeted Therapy of Multiple Liver Metastases After Resected Solitary Gastric Metastasis and Primary Pulmonary Adenocarcinoma. Oncotarget (2016) 7(52):87479–84. doi: 10.18632/oncotarget.13114 PMC5350003 27829227

[25] Del Rosario M Tsai H . Not All Gastric Masses Are Gastric Cancer. BMJ Case Rep (2016) 2016:bcr2015213535. doi: 10.1136/bcr-2015-213535 PMC4800203 26976833

[26] Park JY Hong SW Lee JY Kim JH Kang JW Lee HW . Simultaneous Esophageal and Gastric Metastases From Lung Cancer. Clin Endoscopy (2015) 48(4):332–5. doi: 10.5946/ce.2015.48.4.332 PMC4522427 26240809

[27] Miyazaki J Hirota S Abe T . Metastasis of Lung Cancer to the Gastrointestinal Tract, Presenting With a Volcano-Like Ulcerated Mass. Digest Endoscopy Off J Japan Gastroenterol Endoscopy Soc (2015) 27(3):397–8. doi: 10.1111/den.12412 25491474

[28] Kim MJ Hong JH Park ES Byun JH . Gastric Metastasis From Primary Lung Adenocarcinoma Mimicking Primary Gastric Cancer. World J Gastrointest Oncol (2015) 7(3):12–6. doi: 10.4251/wjgo.v7.i3.12 PMC4357873 25780510

[29] Kim GH Ahn JY Jung HY Park YS Kim MJ Choi KD . Clinical and Endoscopic Features of Metastatic Tumors in the Stomach. Gut Liver (2015) 9(5):615–22. doi: 10.5009/gnl14032 PMC4562778 25473071

[30] Huang Q Su X Bella AE Luo K Jin J Zhang S . Clinicopathological Features and Outcome of Gastric Metastases From Primary Lung Cancer: A Case Report and Systematic Review. Oncol Lett (2015) 9(3):1373–9. doi: 10.3892/ol.2014.2830 PMC4315035 25663915

[31] Gao S Hu XD Wang SZ Liu N Zhao W Yu QX . Gastric Metastasis From Small Cell Lung Cancer: A Case Report. World J Gastroenterol (2015) 21(5):1684–8. doi: 10.3748/wjg.v21.i5.1684 PMC4316115 25663792

[32] Galetta D Catino A Misino A De Ceglie A Logroscino A Simone G . Bladder and Gastric Metastases From Lung Adenocarcinoma Harboring Codon 13 Kras Mutation: A Case Report With Unusual Clinical Outcome. Tumori (2015) 101(5):e138–40. doi: 10.5301/tj.5000358 26045124

[33] Chen CH Chen WM Tung SY Wu CS Tong WL Lee KF . Gastrointestinal Metastasis From Primary Sarcomatoid Carcinoma of the Lung: A Case Report and Review of the Literature. World J Surg Oncol (2015) 13:174. doi: 10.1186/s12957-015-0599-1 25947890 PMC4440284

[34] Chaudhari D Reddy C Young M . Lung Adenocarcinoma With Solitary Gastric Metastasis: A Case Report With Literature Review. Trans Gastrointest Cancer (2015) 4(3):247–51. doi: 10.3978/j.issn.2224-4778.2014.12.03

[35] Hung TI Chu KE Chou YH Yang KC . Gastric Metastasis of Lung Cancer Mimicking an Adrenal Tumor. Case Rep Gastroenterol (2014) 8(1):77–81. doi: 10.1159/000360845 24748862 PMC3985806

[36] Fu SW Yang YH Huang X Yang HX Huang JP . Clinicopathologic Characteristics of Gastric Metastasis From Primary Lung Cancer: A Case Report and Review of the Literature. World Chin J Digestol (2014) 22(18):2657–60. doi: 10.11569/wcjd.v22.i18.2657

[37] Esmadi M Ahmad DS Fu Y Hammad HT . Upper Gastrointestinal Tract Metastasis From Lung Cancer. Digest Liver Dis Off J Ital Soc Gastroenterol Ital Assoc Study Liver (2014) 46(5):474. doi: 10.1016/j.dld.2013.10.019 24315479

[38] Bouzbib C Chaput U Jarrin I Lavergne-Slove A Marteau P Dray X . Bleeding From Gastroduodenal Metastases as the First Manifestation of Lung Adenocarcinoma. Endoscopy (2014) 46 Suppl 1 UCTN:E474–5. doi: 10.1055/s-0034-1377540 25314204

[39] Benedeto-Stojanov D Bjelakovic G Milentijevic M Stojanov D Brzacki V Petrovic G . Metastatic Lesions in the Gastroduodenum - an Unusual Manifestation of Malignant Melanoma and Pulmonary Adenocarcinoma. Cent Eur J Med (2014) 9(6):762–7. doi: 10.2478/s11536-013-0321-z

[40] Kim YI Kang BC Sung SH . Surgically Resected Gastric Metastasis of Pulmonary Squamous Cell Carcinoma. World J Gastrointest Surg (2013) 5(10):278–81. doi: 10.4240/wjgs.v5.i10.278 PMC3812443 24179627

[41] Katsenos S Archondakis S . Solitary Gastric Metastasis From Primary Lung Adenocarcinoma: A Rare Site of Extra-Thoracic Metastatic Disease. J Gastrointest Oncol (2013) 4(2):E11–5. doi: 10.3978/j.issn.2078-6891.2012.057 PMC3635193 23730522

[42] Hu JB Zhu YH Jin M Sun XN . Gastric and Duodenal Squamous Cell Carcinoma: Metastatic or Primary? World J Surg Oncol (2013) 11:204. doi: 10.1186/1477-7819-11-204 23957943 PMC3751751

[43] Diem S Früh M Rodriguez R Liechti P Rothermundt C . Eml4-Alk-Positive Pulmonary Adenocarcinoma With an Unusual Metastatic Pattern: A Case Report. Case Rep Oncol (2013) 6(2):316–9. doi: 10.1159/000352086 PMC3725025 23898275

[44] Sileri P D'Ugo S Del Vecchio Blanco G Lolli E Franceschilli L Formica V . Solitary Metachronous Gastric Metastasis From Pulmonary Adenocarcinoma: Report of a Case. Int J Surg Case Rep (2012) 3(8):385–8. doi: 10.1016/j.ijscr.2012.04.017 PMC3376723 22634567

[45] Jujo T Sakao S Oide T Tatsumi K . Metastatic Gastric Cancer From Squamous Cell Lung Carcinoma. Internal Med (Tokyo Japan) (2012) 51(14):1947–8. doi: 10.2169/internalmedicine.51.7597 22821123

[46] Huang YM Hsieh TY Chen JR Chien HP Chang PH Wang CH . Gastric and Colonic Metastases From Primary Lung Adenocarcinoma: A Case Report and Review of the Literature. Oncol Lett (2012) 4(3):517–20. doi: 10.3892/ol.2012.778 PMC3439000 22970049

[47] Yoshinaga Y Kiyozaki H Okada S Konishi F Yamada S . Granulocyte-Colony-Stimulating Factor-Producing Gastric Metastasis From Large Cell Type Lung Cancer. Clin J Gastroenterol (2011) 4(1):10–4. doi: 10.1007/s12328-010-0196-3 26190614

[48] Wang Y An T Yang L Wang Z Zhuo M Duan J . [Primary Lung Cancer With Gastrointestinal Metastasis: 2 Case Report and Literature Review]. Zhongguo fei ai za zhi = Chin J Lung Cancer (2011) 14(3):278–80. doi: 10.3779/j.issn.1009-3419.2011.03.23 PMC5999648 21426674

[49] Lee PC Lo C Lin MT Liang JT Lin BR . Role of Surgical Intervention in Managing Gastrointestinal Metastases From Lung Cancer. World J Gastroenterol (2011) 17(38):4314–20. doi: 10.3748/wjg.v17.i38.4314 PMC3214707 22090788

[50] Fujiwara A Okami J Tokunaga T Maeda J Higashiyama M Kodama K . Surgical Treatment for Gastrointestinal Metastasis of Non-Small-Cell Lung Cancer After Pulmonary Resection. Gen Thorac Cardiovasc Surg (2011) 59(11):748–52. doi: 10.1007/s11748-011-0811-3 22083693

[51] Trouillet N Robert B Charfi S Bartoli E Joly JP Chatelain D . Gastric Metastases. An Endoscopic Series of Ten Cases. Gastroenterologie clinique biologique (2010) 34(4-5):305–9. doi: 10.1016/j.gcb.2010.01.019 20627637

[52] Özdilekcan Ç Songür N Memiş L Bozdoğ;an N Köksal AŞ Ok U . Lung Cancer Associated With a Single Simultaneous Solitary Metastatic Lesion in Stomach: A Case Report With the Review of Literature. Tuberkuloz ve Toraks (2010) 58(1):78–84. 20517733

[53] Okazaki R Ohtani H Takeda K Sumikawa T Yamasaki A Matsumoto S . Gastric Metastasis by Primary Lung Adenocarcinoma. World J Gastrointest Oncol (2010) 2(10):395–8. doi: 10.4251/wjgo.v2.i10.395 PMC2999676 21160891

[54] Lee MH Kim SR Soh JS Chung MJ Lee YC . A Solitary Gastric Metastasis From Pulmonary Adenocarcinoma: A Case Report. Thorax (2010) 65(7):661–2. doi: 10.1136/thx.2009.122382 20627930

[55] Lo CK Kao SS Tai DKC Ma CC Ho KK Ko KM . Gastrointestinal Metastasis From Primary Lung Cancer. Surg Pract (2009) 13(3):73–6. doi: 10.1111/j.1744-1633.2009.00454.x

[56] Kim MS Kook EH Ahn SH Jeon SY Yoon JH Han MS . Gastrointestinal Metastasis of Lung Cancer With Special Emphasis on a Long-Term Survivor After Operation. J Cancer Res Clin Oncol (2009) 135(2):297–301. doi: 10.1007/s00432-008-0424-0 18512073 PMC12160283

[57] Kanthan R Sharanowski K Senger JL Fesser J Chibbar R Kanthan SC . Uncommon Mucosal Metastases to the Stomach. World J Surg Oncol (2009) 7:62. doi: 10.1186/1477-7819-7-62 19650900 PMC2734526

[58] Guérin E Gilbert O Dequanter D . Acute Abdomen: A Rare Presentation of Lung Cancer Metastasis. Case Rep Med (2009) 2009:903897. doi: 10.1155/2009/903897 19841757 PMC2762241

[59] Facy O Radais F Billard L Chalumeau C Fernoux P Bizollon MH . Gastric Perforation Caused by Metastatic Lung Carcinoma. Am J Surg (2009) 197(1):e5–6. doi: 10.1016/j.amjsurg.2007.12.061 18722581

[60] Aokage K Yoshida J Ishii G Takahashi S Sugito M Nishimura M . Long-Term Survival in Two Cases of Resected Gastric Metastasis of Pulmonary Pleomorphic Carcinoma. J Thorac Oncol Off Publ Int Assoc Study Lung Cancer (2008) 3(7):796–9. doi: 10.1097/JTO.0b013e31817c925c 18594328

[61] Wu MH Lin MT Lee PH . Clinicopathological Study of Gastric Metastases. World J Surg (2007) 31(1):132–6. doi: 10.1007/s00268-006-0177-3 17186432

[62] Rossi G Marchioni A Romagnani E Bertolini F Longo L Cavazza A . Primary Lung Cancer Presenting With Gastrointestinal Tract Involvement: Clinicopathologic and Immunohistochemical Features in a Series of 18 Consecutive Cases. J Thorac Oncol (2007) 2(2):115–20. doi: 10.1016/s1556-0864(15)30037-x 17410025

[63] Li SH Wang SL Huang WT Chiu YC Rau KM . Upper Gastrointestinal Bleeding as the Initial Manifestation of Lung Adenocarcinoma Metastatic to the Stomach. Respir Med Extra (2007) 3(2):67–70. doi: 10.1016/j.rmedx.2007.02.001

[64] Goh BK Yeo AW Koong HN Ooi LL Wong WK . Laparotomy for Acute Complications of Gastrointestinal Metastases From Lung Cancer: Is It a Worthwhile or Futile Effort? Surg Today (2007) 37(5):370–4. doi: 10.1007/s00595-006-3419-y 17468816

[65] Conybeare A Waller DA . Pet Scanning in the Detection of Occult Gastric Metastases From Lung Carcinoma. Eur J Surg Oncol J Eur Soc Surg Oncol Br Assoc Surg Oncol (2007) 33(2):252–3. doi: 10.1016/j.ejso.2006.09.022 17097847

[66] Chang ET Hu Wang A Lin CB Lee JJ Yang GG . Refractory Upper Gastrointestinal Bleeding Occurred in a Patient With Squamous Cell Carcinoma of Lung-A Case Report and Literature Review. Respir Med Extra (2007) 3(1):29–31. doi: 10.1016/j.rmedx.2007.01.005

[67] Yang CJ Hwang JJ Kang WY Chong IW Wang TH Sheu CC . Gastro-Intestinal Metastasis of Primary Lung Carcinoma: Clinical Presentations and Outcome. Lung Cancer (Amsterdam Netherlands) (2006) 54(3):319–23. doi: 10.1016/j.lungcan.2006.08.007 17010474

[68] Ohashi K Kiura K Takigawa N Mizushima T Ino H Tabata M . Successful Treatment of a Patient With Gastric and Duodenal Metastases From Large Cell Carcinoma of the Lung With Carboplatin and Gemcitabine. Anticancer Res (2006) 26(6c):4695–6. 17214328

[69] Casella G Di Bella C Cambareri AR Buda CA Corti G Magri F . Gastric Metastasis by Lung Small Cell Carcinoma. World J Gastroenterol (2006) 12(25):4096–7. doi: 10.3748/wjg.v12.i25.4096 PMC4087731 16810769

[70] Altintas E Sezgin O Uyar B Polat A . Acute Upper Gastrointestinal Bleeding Due to Metastatic Lung Cancer: An Unusual Case. Yonsei Med J (2006) 47(2):276–7. doi: 10.3349/ymj.2006.47.2.276 PMC2687641 16642561

[71] Alpar S Kurt OK Ucar N Orsel O Aydog G Kurt B . A Case of Squamous Cell Lung Carcinoma With Gastric Metastasis. South Med J (2006) 99(11):1313–4. doi: 10.1097/01.smj.0000240697.56774.45 17195443

[72] Kobayashi O Murakami H Yoshida T Cho H Yoshikawa T Tsuburaya A . Clinical Diagnosis of Metastatic Gastric Tumors: Clinicopathologic Findings and Prognosis of Nine Patients in a Single Cancer Center. World J Surg (2004) 28(6):548–51. doi: 10.1007/s00268-004-7216-8 15366743

[73] Nakamura H Mizokami Y Iwaki Y Shiraishi T Ohtsubo T Miura S . Lung Cancer With Metastases to the Stomach and Duodenum. Report of Three Cases. Digest Endoscopy (2003) 15(3):210–5. doi: 10.1046/j.1443-1661.2003.00248.x

[74] Kim HS Jang WI Hong HS Lee CI Lee DK Yong SJ . Metastatic Involvement of the Stomach Secondary to Lung Carcinoma. J Korean Med Sci (1993) 8(1):24–9. doi: 10.3346/jkms.1993.8.1.24 PMC3053845 8393680

[75] Maeda J Miyake M Tokita K Iwahashi N Nakano T Tamura S . Small Cell Lung Cancer With Extensive Cutaneous and Gastric Metastases. Internal Med (Tokyo Japan) (1992) 31(11):1325–8. doi: 10.2169/internalmedicine.31.1325 1338292

[76] Fukuda T Ohnishi Y Katagiri J Ohnuki K Tachikawa S . A Case of Pulmonary Adenocarcinoma With Sarcomatous Elements Initially Manifested as a Submucosal Tumor of the Stomach. Acta Pathol japonica (1992) 42(6):454–9. doi: 10.1111/j.1440-1827.1992.tb03252.x 1502906

[77] Struyf N Lacor P Van den Weyngaert D Bultinck J Mathijs R . Gastric Metastases From Lung Carcinoma. Ann Oncol Off J Eur Soc Med Oncol (1991) 2(9):694–5. doi: 10.1016/S0923-7534(20)30677-3 1660300

[78] O'Donovan MA O'Gorman TA Egan E . Gastric Metastases From Non-Abdominal Primary Malignancies. Irish J Med Sci (1983) 152(4):169–70. doi: 10.1007/bf02960064 6874286

[79] Fletcher MS . Gastric Perforation Secondary to Metastatic Carcinoma of the Lung: A Case Report. Cancer (1980) 46(8):1879–82. doi: 10.1002/1097-0142(19801015)46:8<1879::aid-cncr2820460829>3.0.co;2-a 7427890

[80] Joffe N . Symptomatic Gastrointestinal Metastases Secondary to Bronchogenic Carcinoma. Clin Radiol (1978) 29(2):217–25. doi: 10.1016/s0009-9260(78)80242-6 639463

[81] Michalet JP Faivre J Klepping C . Gastric Metastasis: A Very Uncommon Revealing Manifestation of Bronchial Cancer. J Medecine Lyon (1977) 58(1322):647–50.

[82] Menuck LS Amberg JR . Metastatic Disease Involving the Stomach. Am J Digest Dis (1975) 20(10):903–13. doi: 10.1007/bf01070875 1190198

[83] Edwards R Royle G . Metastatic Carcinoma Causing Haematemesis. Br Med J (1975) 2(5971):598. doi: 10.1136/bmj.2.5971.598 1131630 PMC1673493

[84] Morton WJ Tedesco FJ . Metastatic Bronchogenic Carcinoma Seen as a Gastric Ulcer. Am J Digest Dis (1974) 19(8):766–70. doi: 10.1007/bf01844948 4843220

[85] Yoshimoto A Kasahara K Kawashima A . Gastrointestinal Metastases From Primary Lung Cancer. Eur J Cancer (Oxford Engl 1990) (2006) 42(18):3157–60. doi: 10.1016/j.ejca.2006.08.030 17079136

[86] Bento LH Minata MK Batista CP Martins BD Tolentino LHL Scomparim RC . Clinical and Endoscopic Aspects of Metastases to the Gastrointestinal Tract. Endoscopy (2019) 51(7):646–52. doi: 10.1055/a-0887-4401 31087306

[87] Rosty C Pai RK Graham RP . Clinical and Histological Features of Secondary Carcinomas in Gastrointestinal Tract Biopsies. Histopathology (2020) 77(4):622–30. doi: 10.1111/his.14195 32590886

[88] Antler AS Ough Y Pitchumoni CS Davidian M Thelmo W . Gastrointestinal Metastases From Malignant Tumors of the Lung. Cancer (1982) 49(1):170–2. doi: 10.1002/1097-0142(19820101)49:1<170::aid-cncr2820490134>3.0.co;2-a 6274500

[89] Namikawa T Hanazaki K . Clinicopathological Features and Treatment Outcomes of Metastatic Tumors in the Stomach. Surg Today (2014) 44(8):1392–9. doi: 10.1007/s00595-013-0671-9 23896636

[90] Green LK . Hematogenous Metastases to the Stomach. A Review of 67 Cases. Cancer (1990) 65(7):1596–600. 10.1002/1097-0142(19900401)65:7<1596::aid-cncr2820650724>3.0.co;2-5 2311070

[91] Oda I Kondo H Yamao T Saito D Ono H Gotoda T . Metastatic Tumors to the Stomach: Analysis of 54 Patients Diagnosed at Endoscopy and 347 Autopsy Cases. Endoscopy (2001) 33(6):507–10. doi: 10.1055/s-2001-14960 11437044

[92] Antonini F Laterza L Fuccio L Marcellini M Angelelli L Calcina S . Gastric Metastasis From Ovarian Adenocarcinoma Presenting as a Subepithelial Tumor and Diagnosed by Endoscopic Ultrasound-Guided Tissue Acquisition. World J Gastrointest Oncol (2017) 9(11):452–6. doi: 10.4251/wjgo.v9.i11.452 PMC5700387 29204254

[93] Sekine M Asano T Mashima H . The Diagnosis of Small Gastrointestinal Subepithelial Lesions by Endoscopic Ultrasound-Guided Fine Needle Aspiration and Biopsy. Diagnostics (Basel Switzerland) (2022) 12(4):810. doi: 10.3390/diagnostics12040810 PMC9027519 35453857

[94] Jagirdar J . Application of Immunohistochemistry to the Diagnosis of Primary and Metastatic Carcinoma to the Lung. Arch Pathol Lab Med (2008) 132(3):384–96. doi: 10.5858/2008-132-384-aoittd 18318581

[95] Werling RW Yaziji H Bacchi CE Gown AM . Cdx2, a Highly Sensitive and Specific Marker of Adenocarcinomas of Intestinal Origin: An Immunohistochemical Survey of 476 Primary and Metastatic Carcinomas. Am J Surg Pathol (2003) 27(3):303–10. doi: 10.1097/00000478-200303000-00003 12604886

[96] Reis-Filho JS Carrilho C Valenti C Leitão D Ribeiro CA Ribeiro SG . Is Ttf1 a Good Immunohistochemical Marker to Distinguish Primary From Metastatic Lung Adenocarcinomas? Pathol Res Pract (2000) 196(12):835–40. doi: 10.1016/s0344-0338(00)80084-9 11156325

[97] Santoro R Ettorre GM Santoro E . Subtotal Gastrectomy for Gastric Cancer. World J Gastroenterol (2014) 20(38):13667–80. doi: 10.3748/wjg.v20.i38.13667 PMC4194551 25320505

[98] Chen J Bu Z Ji J . Surgical Treatment of Gastric Cancer: Current Status and Future Directions. Chin J Cancer Res = Chung-kuo yen cheng yen chiu (2021) 33(2):159–67. doi: 10.21147/j.issn.1000-9604.2021.02.04 PMC8181878 34158736

[99] Kosuga T Tsujiura M Nakashima S Masuyama M Otsuji E . Current Status of Function-Preserving Gastrectomy for Gastric Cancer. Ann Gastroenterol Surg (2021) 5(3):278–86. doi: 10.1002/ags3.12430 PMC8164463 34095717

[100] Tan AC Tan DSW . Targeted Therapies for Lung Cancer Patients With Oncogenic Driver Molecular Alterations. J Clin Oncol Off J Am Soc Clin Oncol (2022) 40(6):611–25. doi: 10.1200/jco.21.01626 34985916

[101] Hayashi H Nadal E Gray JE Ardizzoni A Caria N Puri T . Overall Treatment Strategy for Patients With Metastatic Nsclc With Activating Egfr Mutations. Clin Lung Cancer (2022) 23(1):e69–82. doi: 10.1016/j.cllc.2021.10.009 34865963

[102] Soria JC Ohe Y Vansteenkiste J Reungwetwattana T Chewaskulyong B Lee KH . Osimertinib in Untreated Egfr-Mutated Advanced Non-Small-Cell Lung Cancer. New Engl J Med (2018) 378(2):113–25. doi: 10.1056/NEJMoa1713137 29151359

[103] Ramalingam SS Vansteenkiste J Planchard D Cho BC Gray JE Ohe Y . Overall Survival With Osimertinib in Untreated, Egfr-Mutated Advanced Nsclc. New Engl J Med (2020) 382(1):41–50. doi: 10.1056/NEJMoa1913662 31751012

[104] Papini F Sundaresan J Leonetti A Tiseo M Rolfo C Peters GJ . Hype or Hope - Can Combination Therapies With Third-Generation Egfr-Tkis Help Overcome Acquired Resistance and Improve Outcomes in Egfr-Mutant Advanced/Metastatic Nsclc? Crit Rev Oncol/Hematol (2021) 166:103454. doi: 10.1016/j.critrevonc.2021.103454 34455092

[105] Gelatti ACZ Drilon A Santini FC . Optimizing the Sequencing of Tyrosine Kinase Inhibitors (Tkis) in Epidermal Growth Factor Receptor (Egfr) Mutation-Positive Non-Small Cell Lung Cancer (Nsclc). Lung Cancer (Amsterdam Netherlands) (2019) 137:113–22. doi: 10.1016/j.lungcan.2019.09.017 PMC7478849 31568888

