# Supplementary figures and images for: Gastric Metastasis of Primary Lung Cancer: Case Report and Systematic Review With Pooled Analysis
Source: Front Oncol. 2022 Jul 8;12:922016. doi: 10.3389/fonc.2022.922016 (PMC9304872; doi:10.3389/fonc.2022.922016)

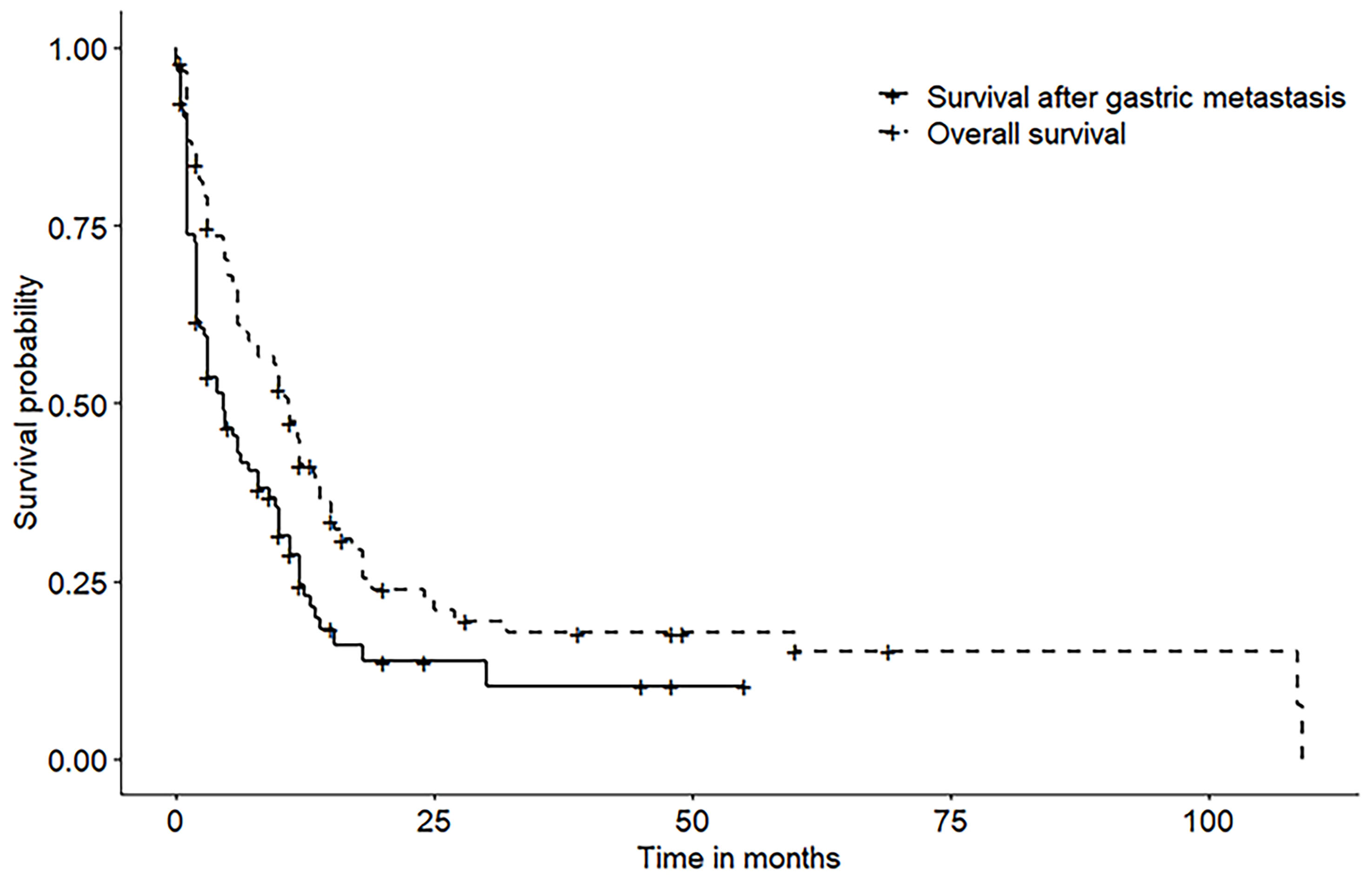

Supplement: Supplementary Figure 1 — Kaplan–Meier plot of the survival after gastric metastasis and overall survival curve. [file Image_1.jpeg]
