# Supplementary material for: Gastric Metastasis of Primary Lung Cancer: Case Report and Systematic Review With Pooled Analysis
Source: Front Oncol. 2022 Jul 8;12:922016. doi: 10.3389/fonc.2022.922016 (PMC9304872; doi:10.3389/fonc.2022.922016)
Supplement: Supplementary file 2 [file DataSheet_1.docx]

**Search strategy**

[PubMed]

(("Lung Neoplasms"[Mesh]) OR ((primary lung carcinoma[Title/Abstract]) OR (primary lung cancer[Title/Abstract]))) AND ((((((((((gastric metastasis[Title/Abstract]) OR (gastric metastases[Title/Abstract])) OR (metastatic gastric carcinoma[Title/Abstract])) OR (metastatic gastric cancer[Title/Abstract])) OR (metastatic gastric tumor[Title/Abstract])) OR (gastrointestinal metastasis[Title/Abstract])) OR (gastrointestinal metastases[Title/Abstract])) OR (secondary gastric cancer[Title/Abstract])) OR (secondary gastric carcinoma[Title/Abstract])) OR (secondary gastric tumor[Title/Abstract]))

Table S1 Clinicopathological Features and Outcome of the 114 Cases

| Author, year | case | Age/gender | Smoking | Primary location | Pathology | Interval* | Other metastasis | Clinical presentation | Gastric location | Endoscopic appearance | Primary treatment | Gastric treatment | Survival# (m) | Overall survival& (m) |
| --- | --- | --- | --- | --- | --- | --- | --- | --- | --- | --- | --- | --- | --- | --- |
| Ours | 1 | 73/M | Y | RUL | AC | Synchronous | Solitary | Abdominal Pain | Fundus | Ulcerated mass | TT | TT | 8+ | 8+ |
| Chang, 2021 | 2 | 55/M | Y | RUL | NSCLC | Metachronous | Solitary | Melena | Corpus | Ulcerated mass | CT | Surgery, CT | 6 | 11 |
| Sgaramella, 2021 | 3 | 59/M | NM | RL | LCC | Synchronous | Multiple | Abdominal Pain | Corpus | Ulcerated mass | CT | Surgery, CT | 3 | 3 |
| Liu, 2021 | 4 | 58/M | NM | RLL | AC | Metachronous | Multiple | Abdominal Pain | Corpus | SMT with ulcer | TT, CRT, Surgery | TT, CT, Surgery | 10+ | 49+ |
| Eiswerth, 2021 | 5 | 61/M | NM | RUL | PLS | Synchronous | NM | Melena | Antrum, Corpus | Mass | NM | NM | NM | NM |
| Duan, 2021 | 6 | 49/F | N | RH | AC | Synchronous | Multiple | Abdominal Distension | Fundus, Corpus | Polypoidal mass | TT | TT | 15+ | 15+ |
| Nemoto, 2020 | 7 | 64/M | Y | RLL | SCC | Metachronous | Solitary | Abdominal Pain, Dysphagia | Cardia | SMT with ulcer | Surgery, CT | Surgery, CT | 13 | 25 |
| Peng, 2019 | 8 | 77/F | N | LH, LLL | SCLC | Synchronous | Multiple | Abdominal Distension, Abdominal Pain | Fundus | NM | CT | CT | 10 | 10 |
| He, 2019 | 9 | 61/M | Y | LLL | SCC | Synchronous | Multiple | Dysphagia | Cardia | SMT with ulcer | Surgery, CT | Surgery, CT | 20+ | 20+ |
| Yang, 2018 | 10 | 59/M | Y | LUL | AC | Synchronous | Multiple | Abdominal Pain | Corpus | Infiltrative ulcerated lesion | CT | CT | NM | NM |
| Nitipir, 2018 | 11 | 66/M | Y | L | NSCLC | Synchronous | Multiple | Abdominal Pain | Fundus | Ulcerated mass | CRT | CRT | 5.5 | 5.5 |
| Li, 2018 | 12 | 61/M | NM | RLL | SCC | Synchronous | Multiple | Abdominal Distension | Corpus | NM | CT | Surgery, CT | 7 | 7 |
| El Hajj, 2018 | 13 | 59/M | NM | RUL | LCC | Metachronous | Solitary | Nausea, Abdominal Pain, Weight Loss | Fundus | Infiltrative ulcerated lesion | Supportive | Supportive | NM | NM |
| Zhang, 2017 | 14 | 61/F | NM | RUL | AC | Metachronous | Multiple | NM | Cardia | SMT | CT | TT | 9.7 | 11.7 |
|  | 15 | 72/M | NM | LH | AC | Synchronous | Solitary | NM | Corpus | Ulcerated nodules | CRT, TT | CRT, TT | 18 | 18 |
| Taira, 2017 | 16 | 64/M | NM | LUL | LCC | Metachronous | Multiple | Melena | Corpus | SMT with ulcer | CT | CT | 6.3 | 13.3 |
|  | 17 | 71/M | NM | LUL | Pleo | Metachronous | Multiple | Melena | Corpus | SMT with ulcer | CT | CT | 4.8 | 10.8 |
| Sharma, 2017 | 18 | 59/M | N | RUL, RML | AC | Synchronous | Solitary | Asymptomatic | Corpus | Polypoidal mass | CT | CT | NM | NM |
| Qasrawi, 2017 | 19 | 69/F | NM | LUL | AC | Metachronous | Multiple | Melena | Corpus | Ulcerated nodules | RT | Supportive | 0.5 | 1.5 |
| Bhardwaj, 2017 | 20 | 39/F | Y | RLL | SCC | Metachronous | Multiple | Melena | Fundus | Ulcerated mass | CT | RT, TT | NM | NM |
| Azar, 2017 | 21 | 90/M | Y | LUL | SCC | Metachronous | Multiple | Melena | Corpus | Ulcer | Supportive | Supportive | 1.8 | 6.8 |
| Ding, 2016 | 22 | 61/F | N | RLL | AC | Metachronous | Multiple | Epigastric Discomfort | Fundus | Ulcerated mass | Surgery | Surgery, CT, TT | 48+ | 48+ |
| Del Rosario, 2016 | 23 | 77/F | NM | L | AC | Synchronous | NM | NM | Whole | Mass | TT | TT | 12+ | 12+ |
| Park, 2015 | 24 | 68/M | NM | LLL | AC | Metachronous | Multiple | Melena, Dysphagia | Whole | Bulging ulcerated lesion | CRT | CT | 4 | 5 |
| Miyazaki, 2015 | 25 | 54/M | NM | RUL | SCC | NM | Multiple | Abdominal Pain, Anemia | Antrum | Bulging ulcerated lesion | Surgery, CT | NM | NM | NM |
| Kim MJ, 2015 | 26 | 68/M | N | LLL | AC | Metachronous | Multiple | Abdominal Pain, Dyspepsia | Fundus | Linitis plastica | Surgery, CT | CT | 15+ | 60+ |
| Kim GH, 2015 | 27 | 44/M | NM | NM | SCC | Metachronous | Solitary | Abdominal Pain | Corpus | Infiltrative ulcerated lesion | NM | surgery | 30 | 60 |
|  | 28 | 55/M | NM | NM | SCLC | Metachronous | Solitary | Bleeding | Corpus | SMT | NM | surgery | 11 | 18 |
|  | 29 | 50/F | NM | NM | SCC | Metachronous | Multiple | Asymptomatic | Whole | Nodules | NM | CT | 0 | 1 |
|  | 30 | 60/M | NM | NM | SCC | Metachronous | Multiple | GI Bleeding | Corpus | Infiltrative ulcerated lesion | NM | Supportive | 2 | 32 |
|  | 31 | 53/M | NM | NM | AC | Synchronous | Solitary | Dysphagia | Corpus | Nodules | NM | Supportive | 6 | 6 |
|  | 32 | 58/M | NM | NM | SCC | Synchronous | Multiple | Asymptomatic | Corpus | Infiltrative ulcerated lesion | NM | Supportive | 1 | 1 |
|  | 33 | 59/M | NM | NM | SCC | Metachronous | Multiple | Asymptomatic | Corpus | SMT | NM | Supportive | 4 | 5 |
| Huang, 2015 | 34 | 61/F | N | RLL | AC | Metachronous | Solitary | Epigastric Discomfort | Fundus | Ulcerated mass | Surgery | Surgery, TT | 5+ | 10+ |
| Gao, 2015 | 35 | 66/M | Y | RH | SCLC | Metachronous | Multiple | Abdominal Pain | Corpus | Ulcer | CRT | CT | 3 | 17 |
| Galetta, 2015 | 36 | 65/F | Y | RH | AC | Metachronous | Multiple | Asymptomatic | Corpus | Ulcerated mass | CT | CT | 9 | 24 |
| Chen, 2015 | 37 | 59/F | N | Both lung | PLS | Synchronous | Multiple | Abdominal Pain | Antrum, Fundus | Ulcerated nodules | Supportive | Supportive | 1 | 1 |
| Chaudhari, 2015 | 38 | 60/F | Y | RUL | AC | Metachronous | Solitary | Hematemesis | Corpus | Infiltrative ulcerated lesion | RT | CT | NM | NM |
| Hung, 2014 | 39 | 47/M | Y | RUL | SCLC | Metachronous | Solitary | Asymptomatic | Corpus | NM | CRT | Surgery, CT | 15+ | 69+ |
| Fu, 2014 | 40 | 60/M | NM | NM | AC | Metachronous | Solitary | Abdominal Pain | Fundus | SMT | Surgery | Surgery, CT | 3+ | 39+ |
| Esmadi, 2014 | 41 | 62/F | Y | LH | LCC | Synchronous | Multiple | Abdominal Pain, Melena | Corpus | Bulging ulcerated lesion | Supportive | Supportive | NM | NM |
| Bouzbib, 2014 | 42 | 64/M | Y | LIL | AC | Synchronous | Multiple | Bleeding, Weight Loss | Fundus | SMT with ulcer | CRT | CRT | 1 | 1 |
| Benedeto-Stojanov, 2014 | 43 | 73/M | Y | NM | AC | Synchronous | Multiple | Melena, Abdominal Pain | Antrum, Cardia | SMT with ulcer | NM | NM | NM | NM |
| Kim, 2013 | 44 | 71/M | Y | RLL | SCC | Synchronous | Multiple | Anemia | Corpus | Infiltrative ulcerated lesion | none | Surgery | 11 | 11 |
| Katsenos, 2013 | 45 | 61/M | Y | LUL | AC | Synchronous | Solitary | Melena | Corpus | Ulcer | CRT | CRT | 10 | 10 |
| Hu, 2013 | 46 | 54/M | Y | RH | SCC | Metachronous | Multiple | Dysphagia | Corpus | Infiltrative ulcerated lesion | Surgery, CRT | none | 2 | 8 |
| Diem, 2013 | 47 | 62/F | N | RUL | AC | Synchronous | Multiple | Abdominal Pain | Cardia | Ulcer | CRT, TT | CRT, TT | 11+ | 11+ |
| Sileri, 2012 | 48 | 68/M | Y | RUL | AC | Metachronous | Solitary | Abdominal Pain | Antrum | SMT | Surgery | Surgery | 9+ | 69+ |
| Jujo, 2012 | 49 | 73/M | NM | RUL | SCC | Synchronous | Multiple | NM | Corpus | Bulging ulcerated lesion | NM | NM | NM | NM |
| Huang, 2012 | 50 | 41/F | Y | LLL | AC | Synchronous | Multiple | Abdominal Pain, Fullness | Antrum | Ulcerated mass | CT, TT | CT, TT | 15.3 | 15.3 |
| Yoshinaga, 2011 | 51 | 73/M | NM | NM | LCC | Metachronous | Solitary | Bleeding | Corpus | Polypoidal mass | Surgery | Surgery | 3+ | 11+ |
| Wang, 2011 | 52 | 71/M | NM | RUL | AC | Metachronous | Multiple | Asymptomatic | Corpus | Polypoidal mass | CRT | CT | 8+ | 13+ |
| Lee，2011 | 53 | 58/F | NM | NM | AC | Metachronous | Multiple | Abdominal Pain | NM | NM | Supportive | Supportive | 15+ | 16+ |
|  | 54 | 79/M | NM | NM | Pleo | Synchronous | Multiple | Hemorrhage | NM | NM | Supportive | Supportive | 1 | 1 |
|  | 55 | 81/M | NM | NM | AC | Metachronous | Multiple | Hemorrhage | NM | NM | Supportive | Supportive | 1 | 6 |
|  | 56 | 73/M | NM | NM | AC | Metachronous | Multiple | Hemorrhage | NM | NM | Supportive | Supportive | 1 | 6 |
|  | 57 | 71/M | NM | NM | SCC | Metachronous | Multiple | Hemorrhage | NM | NM | Supportive | Surgery | 0.5 | 9.5 |
|  | 58 | 59/M | NM | NM | AC | Metachronous | Multiple | Hemorrhage | NM | NM | Supportive | Supportive | 2 | 19 |
|  | 59 | 71/M | NM | NM | SCC | Metachronous | Multiple | Hemorrhage | NM | NM | Supportive | Surgery | 0.5 | 108.5 |
|  | 60 | 70/M | NM | NM | SCLC | Metachronous | Multiple | Abdominal Pain | NM | NM | Supportive | Supportive | 3 | 10 |
| Fujiwara, 2011 | 61 | 51/M | NM | NM | LCC | Metachronous | Multiple | Anemia | NM | NM | Surgery | Supportive | 0.4 | 2.6 |
|  | 62 | 57/M | NM | NM | Pleo | Metachronous | Multiple | Anemia | NM | NM | Surgery | Supportive | 1.1 | 2.3 |
| Trouillet, 2010 | 63 | 65/M | NM | NM | AC | Synchronous | Multiple | Anemia | Antrum | Ulcer | Supportive | Supportive | 2 | 2 |
|  | 64 | 69/M | NM | NM | AC | Metachronous | Solitary | Anemia | Fundus | Bulging ulcerated lesion | Supportive | Supportive | 2 | 3 |
|  | 65 | 74/M | NM | NM | AC | Metachronous | Multiple | Abdominal Pain | Cardia | Bulging ulcerated lesion | Supportive | Supportive | 2 | 3 |
|  | 66 | 54/M | NM | NM | AC | Metachronous | Multiple | Anemia | Antrum | Ulcer | Supportive | Supportive | 1 | 6 |
| Özdilekcan, 2010 | 67 | 46/M | Y | RUL | SCC | Synchronous | Solitary | Dysphagia, Abdominal Pain | Corpus | Ulcer | RT | NM | 1 | 1 |
| Okazaki, 2010 | 68 | 68/M | Y | RLL | AC | Synchronous | Solitary | Abdominal Pain | Corpus | Linitis plastica | CT | CT | 12 | 12 |
| Lee, 2010 | 69 | 77/M | NM | RUL | AC | Synchronous | Solitary | Asymptomatic | Antrum | Bulging ulcerated lesion | Surgery | Surgery | NM | NM |
| Lo, 2009 | 70 | 66/M | Y | NM | SCC | Metachronous | Multiple | Anemia | Corpus | Ulcer | Surgery | NM | 2 | 27 |
|  | 71 | 66/M | Y | NM | AC | Metachronous | Multiple | Anemia | Corpus | Ulcer | Surgery | NM | 1 | 18 |
| Kim, 2009 | 72 | 67/M | N | LUL | AC | Synchronous | Multiple | Abdominal Pain | NM | NM | NM | none | 2.7 | 2.7 |
|  | 73 | 72/M | Y | RUL | LCC | Synchronous | Multiple | Asymptomatic | NM | NM | NM | none | 2.2 | 2.2 |
|  | 74 | 66/M | Y | RLL | SCLC | Synchronous | Multiple | Hematemesis | NM | NM | NM | none | 4.8 | 4.8 |
| Kanthan, 2009 | 75 | 75/M | N | R | AC | Synchronous | NM | Abdominal Pain | NM | Polypoidal mass | NM | NM | NM | NM |
| Guérin, 2009 | 76 | 72/M | NM | LH | SCLC | Synchronous | Multiple | Perforation | NM | Ulcer | CT | Surgery, CT | 8 | 8 |
| Facy, 2009 | 77 | 85/M | Y | RH | SCLC | Synchronous | Multiple | Perforation | Antrum | Ulcer | none | Surgery | 1 | 1 |
| Aokage, 2008 | 78 | 69/M | NM | RUL | Pleo | Metachronous | Solitary | Anemia | Corpus | Ulcerated mass | Surgery | Surgery | 55+ | 60+ |
|  | 79 | 62/M | NM | LUL | Pleo | Metachronous | Solitary | Asymptomatic | Fundus | SMT | Surgery | Surgery | 45+ | 48+ |
| Wu, 2007 | 80 | 73/M | NM | NM | SCC | Metachronous | Multiple | Melena | Cardia | NM | Surgery | Supportive | 1 | 109 |
|  | 81 | 82/M | NM | NM | AC | Metachronous | Multiple | Melena | Antrum | NM | Surgery | Supportive | 1 | 6 |
|  | 82 | 70/M | NM | NM | AC | Metachronous | Multiple | Abdominal Pain | Corpus | NM | Surgery | Supportive | 10 | 15 |
| Rossi, 2007 | 83 | 81/F | Y | NM | LCC | Synchronous | Multiple | Anemia, Abdominal Pain | NM | NM | NM | NM | 1 | 1 |
|  | 84 | 75/M | Y | NM | AC | Synchronous | Solitary | Anemia, GI Bleeding | NM | NM | Surgery, CT | Surgery, CT | 14 | 14 |
|  | 85 | 65/F | N | NM | LCC | Metachronous | Multiple | Anemia, Melena | NM | NM | NM | NM | 2 | 3 |
|  | 86 | 64/F | Y | NM | AC | Synchronous | Multiple | Weight Loss, Pyrosis | NM | NM | CT | CT | 2+ | 2+ |
|  | 87 | 54/M | Y | NM | AC | Synchronous | Multiple | Weight Loss, Abdominal Pain | NM | NM | CT | CT | 0.5+ | 0.5+ |
| Li,2007 | 88 | 49/M | N | RLL | AC | Synchronous | Multiple | Melena | Antrum, Fundus | Infiltrative ulcerated lesion | CT, TT | CT, TT | 12 | 12 |
| Goh, 2007 | 89 | 54/M | NM | LUL | Pleo | Metachronous | Solitary | Hemorrhage | NM | NM | Surgery | Surgery | 8 | 16 |
|  | 90 | 70/M | NM | LUL | SCC | Synchronous | Multiple | Hemorrhage | NM | NM | none | Surgery | 0.6 | 0.66 |
| Conybeare, 2007 | 91 | 76/M | Y | LUL | NSCLC | Synchronous | Multiple | NM | Corpus | Ulcerated mass | NM | NM | NM | NM |
| Chang, 2007 | 92 | 52/M | Y | RUL | SCC | Synchronous | Solitary | Melena | Corpus | SMT with ulcer | CRT | CRT | NM | NM |
| Yang, 2006 | 93 | 71/M | Y | LUL | SCC | Synchronous | Solitary | Melena | NM | NM | NM | NM | 4.5 | 4.5 |
|  | 94 | 65/M | Y | RML | SCC | Synchronous | Multiple | Melena | NM | NM | NM | NM | 3 | 3.03 |
|  | 95 | 62/M | Y | RUL | AC | Metachronous | Multiple | Melena | NM | NM | NM | NM | 12.4 | 14 |
| Ohashi, 2006 | 96 | 62/M | NM | RUL | LCC | Metachronous | Multiple | Abdominal Pain | Fundus | Ulcerated mass | Surgery, CT | Surgery, CT | 24+ | 28+ |
| Casella, 2006 | 97 | 63/M | Y | LH | SCLC | Synchronous | Multiple | Abdominal Pain | Corpus | Bulging ulcerated lesion | Supportive | Supportive | 1 | 1 |
| Altintas, 2006 | 98 | 55/M | NM | NM | AC | Metachronous | Multiple | Melena, Hematemesis | Corpus | Bulging ulcerated lesion | CRT | Supportive | 0.75 | 11.75 |
| Alpar, 2006 | 99 | 66/M | Y | RUL | SCC | Metachronous | Multiple | Abdominal Pain, Vomiting | NM | Erosive and atrophic pangastritis | CT | none | 2 | 10.5 |
| Kobayashi, 2004 | 100 | 51/M | NM | NM | LCC | Synchronous | Solitary | Dysphagia | Corpus | Bulging ulcerated lesion | Surgery | Surgery, CT | 13.5 | 13.5 |
|  | 101 | 65/M | NM | NM | SCLC | Synchronous | Multiple | Abdominal Pain | Corpus | SMT | CT | Surgery, CT | 4.5 | 4.5 |
| Nakamura, 2003 | 102 | 74/M | NM | RUL | AC | Metachronous | Multiple | Melena, Anemia | Corpus | SMT with ulcer | CT | NM | NM | NM |
| Kim, 1993 | 103 | 66/M | NM | LH | SCLC | Synchronous | Multiple | Abdominal Pain | Corpus, Fundus | SMT with ulcer | NM | NM | NM | NM |
|  | 104 | 68/M | Y | LH | SCC | Synchronous | Multiple | Asymptomatic | Corpus | Polypoidal mass | NM | NM | NM | NM |
| Maeda, 1992 | 105 | 60/F | NM | RLL | SCLC | Metachronous | Multiple | Nausea, Vomiting | Whole | SMT | CT | none | NM | NM |
| Fukuda, 1992 | 106 | 79/F | NM | RLL | AC | Metachronous | Multiple | Abdominal Pain | Fundus | SMT with ulcer | NM | NM | 12 | 12 |
| Struyf, 1991 | 107 | 72/M | NM | RUL | LCC | Synchronous | Multiple | Hemorrhage | Whole | Nodules | Supportive | Supportive | 0 | 0 |
| O'Donovan, 1983 | 108 | 73/M | NM | RH | SCLC | Synchronous | NM | Abdominal Pain, Fullness | Corpus | SMT with ulcer | CT | CT | NM | NM |
| Fletcher, 1980 | 109 | 70/M | Y | LLL | SCC | Synchronous | Solitary | Perforation | Corpus | Ulcerated mass | none | Surgery | 2 | 2 |
| Joffe, 1978 | 110 | 63/M | NM | RH | SCLC | Metachronous | NM | Epigastric Distress, Haematemesis | Antrum | Ulcerated mass | NM | NM | NM | NM |
|  | 111 | 66/F | NM | LUL | AC | Metachronous | NM | Abdominal Pain | Fundus | SMT with ulcer | Surgery | NM | NM | NM |
| Menuck, 1975 | 112 | 48/M | NM | NM | SCC | Metachronous | NM | Nausea, Abdominal Pain, Melena | Corpus | Linitis plastica | NM | NM | 3 | 15 |
| Edwards, 1975 | 113 | 59/M | Y | LUL | SCLC | Synchronous | Multiple | Haematemesis | Corpus | Ulcer | NM | NM | 0.13 | 0.13 |
| Morton,1974 | 114 | 74/F | Y | LUL | SCC | Synchronous | Multiple | Abdominal Pain, Nausea, Vomiting | Corpus | Ulcerated mass | RT | RT | 3+ | 3+ |

*Interval, time between primary lung tumor diagnosis and Gastric metastasis; #, Survival after diagnosis of metastatic gastric cancer; &, Survival after diagnosis of primary lung cancer; +, alive; M, male; F, female; NM, not mentioned; AC, adenocarcinoma; SCC, squamous cell carcinoma; LCC, large cell carcinoma; SCLC, small cell lung carcinoma; NSCLC, nonsmall cell lung carcinoma; PLS, primary lung sarcoma; Pleo, pleomorphic carcinoma; RUL, right upper lobe; RML, right middle lobe; RLL, right lower lobe; RH, right hilum; LUL, left upper lobe; LLL, left lower lobe; LH, left hilum; R, right lung; L, left lung; SMT, submucosal tumor; CRT, chemoradiotherapy; CT, chemotherapy; RT, radiotherapy; TT, targeted therapy;
